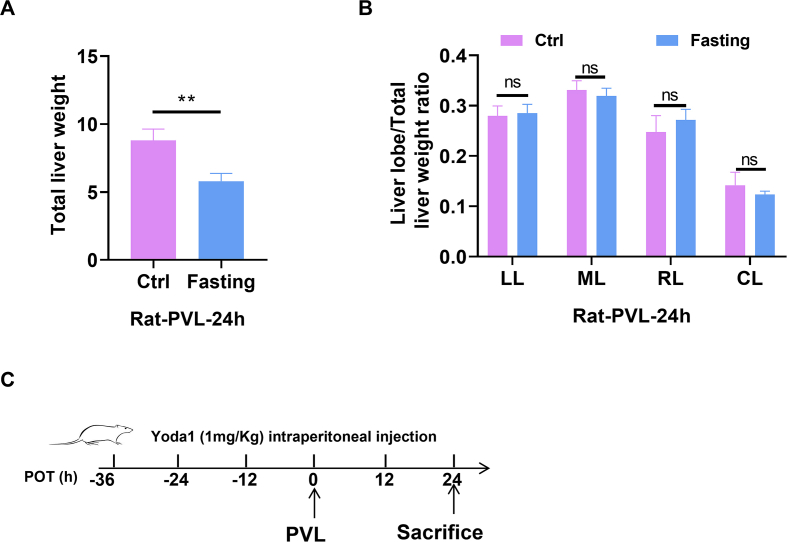# EGFR-mediated crosstalk between vascular endothelial cells and hepatocytes promotes Piezo1-dependent liver regeneration

**DOI:** 10.1016/j.gendis.2024.101321

**Published:** 2024-05-08

**Authors:** Yuelei Hu, Guifang Du, Chao Li, Rui Wang, Juan Liu, Yunfang Wang, Jiahong Dong

**Affiliations:** a Department of Hepatobiliary and Pancreatic Surgery, The First Hospital of Jilin University, Jilin University, Changchun, Jilin 130021, China; b Research Unit of Precision Hepatobiliary Surgery Paradigm, Chinese Academy of Medical Sciences, Beijing 102218, China; c Hepatopancreatobiliary Center, Beijing Tsinghua Changgung Hospital, School of Clinical Medicine, Tsinghua University, Beijing 102218, China; d Department of Biomedical Engineering, School of Medicine, Tsinghua University, Beijing 100084, China; e Institute for Organ Transplant and Bionic Medicine, Tsinghua University, Beijing 100084, China; f Clinical Translational Science Center, Beijing Tsinghua Changgung Hospital, Tsinghua University, Beijing 102218, China

**Keywords:** EGFR, Hemodynamic changes, Liver regeneration, Piezo1, Vascular endothelial cells

## Abstract

Hepatocyte proliferation is essential for recovering liver function after injury. In liver surgery, the mechanical stimulation induced by hemodynamic changes triggers vascular endothelial cells (VECs) to secrete large amounts of cytokines that enhance hepatocyte proliferation and play a pivotal role in liver regeneration (LR). Piezo1, a critical mechanosensory ion channel, can detect and convert mechanical forces into chemical signals, importing external stimuli into cells and triggering downstream biological effects. However, the precise role of Piezo1 in VECs, especially in terms of mediating LR, remains unclear. Here, we report on a potential mechanism by which early changes in hepatic portal hemodynamics activate Piezo1 in VECs to promote hepatocyte proliferation during the process of LR induced by portal vein ligation in rats. In this LR model, hepatocyte proliferation is mainly distributed in zone 1 and zone 2 of liver lobules at 24–48 h after surgery, while only a small number of Ki67-positive hepatocytes were observed in zone 3. Activation of Piezo1 promotes increased secretion of epiregulin and amphiregulin from VECs via the PKC/ERK1/2 axis, further activating epidermal growth factor receptor (EGFR) and ERK1/2 signals in hepatocytes and promoting proliferation. In the liver lobules, the expression of EGFR in hepatocytes of zone 1 and zone 2 is significantly higher than that in zone 3. The EGFR inhibitor gefitinib inhibits LR by suppressing the proliferation of hepatocytes in the middle zone. These data provide a theoretical basis for the regulation of LR through chemical signals mediated by mechanical stimulation.

## Introduction

The liver is an “injury-privileged” organ that can regenerate and recover after partial hepatectomy within a defined period, despite the loss of up to two-thirds of liver parenchyma.^1^ Nonetheless, acute liver failure is a life-threatening postoperative complication that can occur due to the substantial loss of liver parenchyma and is a significant limiting factor for liver surgery. Therefore, strategies aimed at promoting liver regeneration (LR) and recovery are crucial for overcoming the rate-limiting steps in the field of liver surgery.^2^^,^^3^ LR is a complex biological process involving the proliferation of parenchymal (hepatocytes) and non-parenchymal cells within the liver. The process involves a complex network of inflammatory and growth factors.^1^^,^^4^ Transient epithelial-mesenchymal transition-like (EMT-like) response is an important step in LR. The interaction between transforming growth factor-β1 and Hippo-Yap signaling pathways stimulates hepatocytes to undergo EMT-like responses, which are necessary for them to grow and regenerate damaged livers in a transforming growth factor-β1-rich microenvironment.^5^ At present, there are few studies on EMT-like response during LR, and the mechanism is not fully understood. Overcoming the challenges associated with liver surgery requires an understanding of the molecular mechanisms that govern LR and identifying reliable strategies to optimize and augment LR.

Hemodynamic changes resulting from liver surgery disrupt the mechanical equilibria of cells within the liver, especially VECs.^6^ Consequently, the relationship between VECs and mechanical forces has become an increasingly important area of study for LR.^7^ Recent research indicates that hemodynamic changes play a critical role as “triggers and balancers” in the induction of LR following the loss of liver parenchyma.^6^ Specifically, the mechanical effects of hemodynamic changes on VECs are key to initiating and regulating LR.^8^ Portal vein embolization, for example, leads to increased tensile forces on VECs, which triggers the production of high levels of interleukin-6, a key factor previously implicated in initiating LR.^9^ Moreover, increased portal vein blood flow induces the expression of hepatocyte growth factor by VECs, which promotes hepatocyte proliferation and hypertrophy, reduces apoptosis, and facilitates LR.^10^ Blocking the increase in portal vein blood flow significantly delays LR.^6^^,^^10^ Therefore, the stimulation of hemodynamic changes in the process of LR represents a crucial mechanism for initiating and regulating the regenerative response.^4^

Cells perceive mechanical forces predominantly through mechanosensing structures found on the cell surface.^4^ Piezo1 activation in liver sinusoidal endothelial cells promotes the production of CXCL1 and the formation of neutrophil trapping nets that lead to microvascular microthrombi formation and accelerated development of liver cirrhosis in Budd-Chiari syndrome.^11^ The transient effects of hemodynamic changes induced by partial hepatectomy, portal vein ligation (PVL), and associated liver partition and portal vein ligation for staged hepatectomy on VECs may differ from phenomena observed in Budd-Chiari syndrome. Furthermore, the role of Piezo1 in postoperative LR and its underlying mechanisms remain unclear. Therefore, in this study, we investigate the regulatory effects of Piezo1 activation in VECs on hepatocyte proliferation, and whether this activation could be a potential target for improving LR.

## Materials and methods

### Rat PVL model

Sprague–Dawley rats weighing approximately 200–240 g were procured from Charles River Laboratories (Beijing, China). All experimental protocols were approved by the Ethics Committee of Beijing Charles River Laboratory Animal Technology. To investigate the pattern and regularity of LR following 70% PVL, we randomly divided 28 rats into seven groups of four rats each: sham-operated, 12, 24, 48, 72, 120, and 168 h post-surgery. The surgical procedure was performed as previously described.^12^ Briefly, rats were anesthetized using isoflurane, and an incision was made in the anterior midline of the abdominal wall. The portal vein was carefully separated from the bile duct and hepatic artery at the location of the main trunk of the portal vein entry into the left and middle liver, and double-ligated with 4–0 silk suture. To explore the effect of gefitinib on PVL-induced LR, six additional rats were randomly divided into two groups of three each. Rats in the two groups received intraperitoneal administration of gefitinib (20 mg/kg) or the vehicle only, respectively, every 12 h starting 36 h before PVL and continuing until 48 h after PVL. Liver tissue samples were collected from the regenerated liver of rats. To investigate the impact of fasting and feeding on LR, six Sprague–Dawley rats were divided into two groups. After a 12-h fasting period, a 70% PVL was performed. After the operation, the control group was allowed unrestricted access to food, while the fasting group continued fasting for an additional 24 h. Samples were collected for subsequent experiments. In a separate study on the influence of Yoda1 on liver regeneration under fasting conditions, seven rats were divided into two groups: three in the control group and four in the Yoda1 group. The groups were treated with vehicle and Yoda1 (1 mg/kg), respectively. Specimens were collected 24 h after fasting and surgical treatment for further analysis.

We have previously reported the detailed analysis process and results of ultrasound localization microscopy on microvascular blood flow changes after PVL.^13^ Briefly, the abdomen of the rat was shaved to provide an acoustic imaging window and sterilized using 70% alcohol. With the animal fixed in the supine position on a heating pad, a bolus of 0.2 mL of 5-fold diluted Supervue MBs (NUMT, Nanjing, China) was injected through the femoral vein. Ultrasound localization microscopy data acquisition was performed longitudinally at two time points for three rats: a baseline scan performed before PVL and an acute response scan performed 30 min after PVL. Detailed processing was described in our published report.^13^ To assess the impact of fasting on portal blood flow, four Sprague–Dawley rats were randomly assigned into two groups. After a 12-h fasting period, 70% PVL was performed. The postoperative control group was provided with a normal diet, while the fasting group continued fasting with access only to water. Portal blood flow measurements were conducted 18 h after PVL.

### Isolation of primary hepatocytes and endothelial cells, cell lines, and cell culture

The 8-week-old male mice were placed on the table after respiratory anesthesia, and the inferior vena cava and hepatic portal vein trunk were exposed after a cross incision in the abdomen. The 22G indwelling needle was fixed with 4–0 silk thread after puncture of the main portal vein, and the peristaltic pump was kept at 2 mL/min for solution 1 perfusion. At the same time, the inferior vena cava was opened for blood drainage. When there was no obvious blood outflow in the mice, the solution was replaced with solution 2, the speed remained unchanged, and the perfusion lasted about 10 min. The solution was then replaced with solution 3, and the perfusion was continued for 10 min. Subsequently, 0.2 mg/mL collagenase IV solution was replaced for perfusion, and the perfusion was terminated according to the digestive state of the liver. Subsequently, the liver tissue was taken out and the liver cells were isolated in the pre-cooled Dulbecco's modified Eagle medium (DMEM). The precipitated cells were collected after centrifugation at 50*g* for 3 min and re-suspended and again centrifuged for 3 times to obtain primary hepatocytes. The supernatant was collected and centrifuged at 50 *g* for 5 min. The supernatant was centrifuged at 500 *g* in another centrifuge tube for 5 min, and centrifuged three times after resuspension to obtain endothelial cells for culture. Rat tail collagen was used to coat the cell culture plate before cell application. The formulation of solutions 1–3 is available in Table S1.

Human umbilical vein endothelial cells (HUVEC) and SK-Hep1^14^ cells were immortalized and cultured in DMEM supplemented with 10% fetal bovine serum and 1% penicillin-streptomycin at 37 °C in a 5% CO_2_ incubator. Human hepatocyte cell lines LO2 and HepaRG were also cultured under the same conditions.

## Compounds and proteins

Yoda1 (CAS No. 448947-81-7), gefitinib (CAS No. 184475-35-2), ravoxertinib (CAS No. 1453848-26-4), adezmapimod (SB 203580) (CAS No. 152121-47-6), SR11302 (CAS No. 160162-42-5), SP600125 (CAS No. 129-56-6), and GO 6983 (CAS No. 133053-19-7) were purchased from MedChemExpress (Monmouth Junction, NJ, USA). Staurosporine (AM-2282) (CAS: 62996-74-1) was purchased from Selleck Chemicals (Houston, TX, USA). Recombinant human amphiregulin (AREG) and epiregulin (EREG) proteins (262-AR-100 and 1195-EP-025) were purchased from R&D Systems (Minneapolis, MN, USA). AREG and EREG proteins (HY–P77868 and HY-P75226) were purchased from MedChemExpress.

### Immunofluorescence staining and immunohistochemistry

Liver tissue samples were fixed with formaldehyde, embedded in paraffin blocks, and sectioned at 4 μm. Immunofluorescence staining for the protein Ki67, proliferating cell nuclear antigen (PCNA), and epidermal growth factor receptor (EGFR) was done; antigen retrieval was performed using 10 mM sodium citrate (pH 6.0). Non-specific binding was blocked using 5% donkey serum at room temperature for 1 h. Liver tissue sections were incubated with primary antibodies of Ki67 (Abcam ab15580, 1:200), PCNA (Abcam ab29, 1:100), and EGFR (Abcam, ab52894 1:50) at 4 °C overnight, and then with corresponding secondary antibodies at room temperature for 1 h. Nuclei were stained with DAPI (blue) for 15–30 min. Finally, images were captured using 3DHistech software.

For cell immunofluorescence, cells were plated at a density of 4000 cells per well in a 96-well plate and allowed to attach overnight. After experimental treatment, cells were fixed with 4% paraformaldehyde at room temperature for 15 min and then permeabilized with 0.3% Triton in Tris-buffered saline with Tween-20 (TBST) for 10 min. Non-specific binding was blocked with 5% donkey serum at room temperature for 1 h. Cells were then incubated with PKCα and Ki67 (PKCα, CST: #2056, 1:50 diluted in 5% donkey serum and 0.3% Triton X-100 in TBST; Ki67, Abcam ab15580, 1:200) at 4 °C overnight. After washing with phosphate buffer saline (PBS) for 5 min in triplicate, the cells were incubated with the secondary antibody at room temperature for 1 h. Nuclei were stained with DAPI for 15–30 min, and then cells were again washed three times with TBST for 5 min each. A high content analysis system was used to photograph and image the cells after adding 50 μL of PBS per well.

For immunohistochemistry, after the slice antigen retrieval was performed, the slices were placed in 3% hydrogen peroxide for 10 min to deactivate endogenous peroxidases. After washing with Tris-buffered saline (TBS), non-specific binding was blocked with 5% horse serum at room temperature for 1 h. Following TBS washing, avidin solution was applied and left at room temperature for 15 min (Vector Lab Avidin/Biotin Blocking Kit #SP-2001). After TBS washing, biotin solution was applied and left at room temperature for 15 min (Vector Lab Avidin/Biotin Blocking Kit #SP-2001). Primary antibodies against Ki67 (Abcam ab15580, 1:1000) was diluted in blocking solution and incubated at 4 °C overnight. Following TBS washing, secondary antibodies (VECTASTAIN Elite ABC universal Kit peroxidase #PK-6200) were applied and incubated at room temperature for 60 min. After washing, A and B reagents in the ABC kit were incubated at room temperature for 60 min. Subsequently, color development was initiated using the Vector Novared Substrate Kit Peroxidase #SK-4800. After hematoxylin counterstaining and air-drying of the slides, they were dehydrated and sealed with neutral resin. Finally, images were captured using 3D Histech software.

### Establishment of VEC cell lines with stable Piezo1 knockdown

Lentiviral particles (hU6-MCS-CBh-gcGFP-IRES-puromycin) containing Piezo1 shRNA (No.09 sense: 5′-gcACTCCATTATGTTCGAGGA-3′, No.10 sense: 5′-gaAGACCACATTC AGGTGGAA-3′, No.11 sense: 5′-ccCTGTGCATTGATTATCCCT-3′) were purchased from GeneChem (Shanghai, China). Recombinant lentiviral particles were used to infect HUVEC and SK-Hep1 cells according to the manufacturer's instructions. Stably infected cell lines were selected using puromycin at a concentration of 5 μg/mL.

### Western blot (WB) analysis

Proteins were extracted from cell lines using a radioimmunoprecipitation assay buffer with a protease and phosphatase inhibitor cocktail (Beyotime, China). Proteins were separated using a 10% SDS-PAGE gel and transferred to a PVDF membrane using the wet transfer method at 120V for 1–2 h. For Piezo1 transfer, a special method was performed with 240 mA and 3.5 h using a transfer buffer containing 1% SDS and 10% methanol. The membrane was then blocked with 5% fat-free milk at room temperature for 1 h. Primary antibodies were added and incubated at 4 °C overnight (48 h for Piezo1). After washing the membrane with TBST buffer for 5 min in triplicate, an HRP-conjugated secondary antibody was added at a dilution of about 1:1000 for another 2-h incubation at room temperature. Bands were detected using an enhanced chemiluminescence reagent (Applygen, China). GAPDH and β-actin was used as internal references. The antibodies used for WB testing included anti-Piezo1 (Proteintech #15939-1-AP, 1:500), anti-p-EGFR (ABclonal #AP0820, 1:1000), anti-EGFR (Proteintech #8986-1-AP AP0820, 1:1000), anti-ERK1/2 (CST #4695, 1:1000), anti-p-ERK1/2 (CST #4376, 1:1000), anti-PKCα (CST #2056, 1:1000), anti-CyclinD1 (Abcam ab134175, 1:1000), anti-E-cadherin (Proteintech, 20874-1-AP1:1000), anti-vimentin (Abcam, ab92547, 1:1000), anti-GAPDH (Servicebio GB15004, 1:1000), and anti-β-actin (Servicebio GB11001, 1:1000).

### Quantitative reverse transcription PCR (qRT-PCR)

Total RNA was extracted from cell lines of different groups using TRIzol reagent (Invitrogen, Carlsbad, CA, USA), and cDNA was synthesized with a reverse transcription kit (Toyobo FSQ 301, Japan) following the manufacturer's protocol. The mRNA expression levels of target genes were measured using a quantitative fluorescence kit (Toyobo QPS-201, Japan) and relative RNA expression levels were normalized to β-actin (human) or GAPDH (mouse). The 2^−ΔΔCt^ method was used to analyze relative gene expression levels. The primers used in this study were synthesized by Ruibiotech (Beijing, China) and their sequences are provided in Table S2.

### Preparation of conditioned medium

To prepare conditioned medium (CM), VECs (HUVEC and SK-Hep1) were seeded into 10-cm dishes at approximately 30%–40% density. After overnight incubation, fetal bovine serum-free DMEM containing Yoda1 (10 μM) or vehicle was added according to experimental requirements to activate Piezo1 of the VECs for approximately 5–6 h. To exclude a potential non-specific effect of Yoda1, cells were then washed three to five times with PBS and the medium changed to fetal bovine serum-free DMEM. Cells were then incubated for 20–24 h and then collected and centrifuged at 2000 rpm at 4 °C for 10 min. The supernatant was retained as CM and used to culture hepatocytes, with the addition of drugs according to experimental requirements. Six CMs were produced: HCM (conditioned media from HUVEC with DMEM), HCM^DMSO^ (conditioned medium from HUVEC with treatment by DMSO), HCM^Yoda1^ (conditioned media from HUVEC with Piezo1 activated by Yoda1), SCM (conditioned medium from SK-Hep1 with DMEM), SCM^DMSO^ (conditioned medium from SK-Hep1 with treatment by DMSO), and SCM^Yoda1^ (conditioned medium from SK-Hep1 with Piezo1 activated by Yoda1).

### Cell proliferation assay

Hepatocytes were subjected to different experimental conditions, and then 3-(4,5-dimethylthiazol-2-yl)-2,5-diphenyltetrazolium bromide (MTT) solution was added to the culture medium with a working concentration of 5 mg/mL. After 6 h of incubation, the upper medium was removed, and crystals formed in the lower medium were dissolved using 150 μL DMSO. After 10 min of shaking, absorbance was measured at a wavelength of 490 nm.

EdU cell proliferation assay kit (Cell-Light EdU Apollo567 In Vitro Kit (100T), C10310-1, RIBOBIO) was used to detect the proliferation of primary mouse hepatocytes. Finally, the high content analysis system was used to take pictures.

### Intracellular calcium ion detection

Fluo-4AM calcium detection kit (Beyotime, S1061S) was used to detect intracellular calcium levels. Briefly, HUVEC and SK-Hep1 cells were placed in a 96-well plate at 8000/well. First, 1 × Fluo-4AM probe was loaded and incubated at 37 °C for 30 min. Subsequently, different concentrations of Yoda1 were added to detect the fluorescence intensity on a fluorescence microplate reader for 1, 5, 10, 15, and 30 min, respectively. The excitation light was 488 nm and the emission light was 528 nm. Subsequently, the high content analysis system was used to take pictures.

### Cell morphology observation

Hepatocytes subjected to different experimental conditions were fixed with absolute methanol for 15 min, washed with PBS three times for 5 min each, and stained with crystal violet for 10–15 min. The cells were again triple-washed with PBS for 5 min, and their morphological changes were recorded by microscopy after adding PBS.

### RNA sequencing assay

When HUVEC and SK-Hep1 cells plated in 10-cm cell culture dishes reached 30%–40% confluence, culture dishes were treated with 10 μM Yoda1 in fetal bovine serum-free DMEM for 6 h. Three independent samples of two cell lines in each group (DMSO and Yoda1) were used for RNA sequencing by Annoroda Gene Technology (Beijing, China). To understand the potential signal pathway of Yoda1-induced gene expression in HUVEC and SK-Hep1 cell lines, we performed gene set enrichment analysis (GSEA) using software obtained online (http://www.gsea-msigdb.org/gsea/index.jsp). To gain further insight into Yoda1-induced differences in gene expression changes, the online website bioladder (https://www.bioladder.cn/web/#/pro/cloud) was used to analyze gene expressions in the two cell lines of different groups. Log2[mRNA fold change] was used to identify differentially expressed mRNAs, with the calculated value of < −1.5 or >1.5 deemed statistically significant (*P* ≤ 0.05). The online bioinformatics database (DAVID Bioinformatics Resources 6.8, NIAID/NIH, https://david.ncifcrf.gov/tools.jsp) was used to analyze the Kyoto Encyclopedia of Genes and Genomes (KEGG) pathways based on the up-regulated gene list. The human ligand–receptor interaction pairs list was obtained from the website CellTalkDB (http://tcm.zju.edu.cn/celltalkdb/download.php). The overlapping gene lists of up-regulated genes in the Yoda1-induced group of HUVEC and SK-Hep1 cell lines with ligand genes were again subjected to KEGG analysis using the DAVID website.

### Enzyme-linked immunosorbent assay (ELISA)

Approximately 4 × 10^5^ cells were seeded into a six-well plate overnight and incubated with Yoda1 to induce Piezo1 activation. The supernatant from VECs was collected and centrifuged to remove particles for further analysis. Levels of AREG and EREG were measured using AREG (EK0304) and EREG (EK1394) ELISA kits from Boster Biological Technology (Wuhan, China) following the manufacturer's protocol. Samples were run in duplicate and three independent experiments were conducted.

### Statistical analysis

All experimental data were presented as mean ± standard error from at least three independent experiments. Statistical analyses were performed using GraphPad Prism 8 software. To compare data between two different treatment groups, a two-tailed, unpaired Student's *t*-test was used. Differences were considered statistically significant when *P* ≤ 0.05.

## Results

### Portal vein hemodynamic alterations accompany PVL-induced regional LR in rats and Piezo1 is highly expressed in VECs

To investigate the impact of changes in portal vein blood flow on LR, we used a rat PVL model. Figure 1A illustrates the experimental setup and surgical procedure. Initially, PVL leads to liver tissue atrophy on the ligated side and regeneration on the non-ligated side (Fig. S1A). The regenerated liver tissue regained approximately 90% of the whole liver weight in 120–168 h (Fig. 1B). During early regeneration stages, the distribution of Ki67-positive hepatocytes was zone-dependent. Specifically, Ki67-positive hepatocytes were concentrated primarily in zone 1 and zone 2, with a significantly lower proportion observed around central veins (Fig. 1C; Fig. S1B). Notably, in zone 1, Ki67-positive hepatocytes increased rapidly at 24–72 h, followed by a gradual decrease (Fig. 1D; Fig. S1C).

**Fig. 1:**
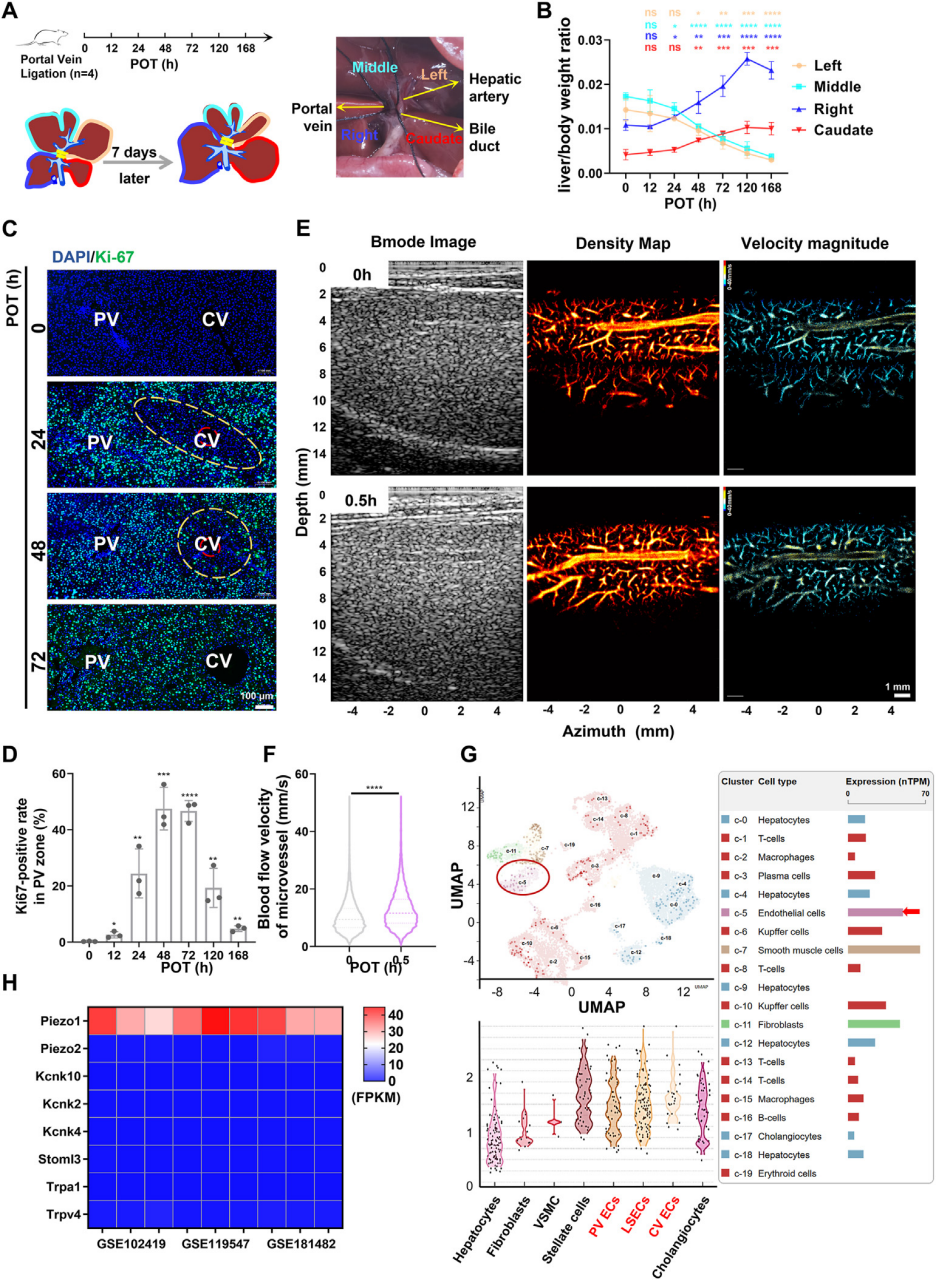
Distribution characteristic of proliferating hepatocytes and hemodynamic changes in PVL-induced liver regeneration and expression of Piezo1 in various liver cells. **(A)** Schematic diagram of the rat PVL model. **(B)** Changes in the ratio of liver weight to body weight in each lobe after 70% PVL in rats (the changes of each liver lobe were compared with 0 h). **(C)** Immunofluorescence staining analysis of Ki67 (in PV-CV) in paraffin tissues from the right liver lobe at 0, 24, 48, and 72 h after 70% PVL. Scale bar: 100 μm. **(D)** Ki67-positive hepatocyte changes during liver regeneration in the PV zone, Ki67-positive hepatocytes were counted in three randomly chosen PV fields, and the average count of these three fields was set as the Ki67-positive cell number of one rat (the statistical analysis of each time point was obtained by comparing with the results of 0 h). **(E, F)** Ultrasound localization microscopy assessment of hepatic microvascular blood flow changes between 0 h and 0.5 h after PVL. **(G, H)** The expression level of Piezo1 (from single-cell RNA sequencing) in different cell subsets in the human liver (https://www.proteinatlas.org/, https://livercellatlas.org) and mouse LSECs (GEO data: GSE102419, GSE119547, and GSE181482). ∗*P* < 0.05, ∗∗*P* < 0.01, ∗∗∗*P* < 0.001, ∗∗∗∗*P* < 0.0001; two-tailed Student's *t*-tests. PVL, portal vein ligation; LL, left lobe; ML, middle lobe; RL, right lobe; CL, caudal lobe; POT, post-operative time; PV, portal vein; CV, central vein; LSEC, liver sinusoidal endothelial cell.

We explored the hemodynamic changes of microvascular blood flow after PVL using ultrasound localization microscopy. Postoperative microvascular blood flow velocity significantly increased at 30 min after PVL (Fig. 1E, F). Piezo1, an important mechanically activated ion channel responsive to various mechanical stimuli such as shear stress and tension, is expressed in many tissues, including liver tissue (Fig. S2A). To clarify the expression level of Piezo1 in different liver cell types, we queried its expression in different liver cells through multiple searchable single-cell sequencing databases. Expression levels of Piezo1 were high in VECs (Fig. 1G; Fig. S2B, C). Additionally, high expression of Piezo1 in liver VECs of mice, pigs, and monkeys was found (Fig. S2D). Cell sequencing of liver sinusoidal endothelial cells in the GEO database suggested that Piezo1 expression was higher than other mechanically activated ion channels (Fig. 1H). Therefore, we further explored the effect of Piezo1 activation in VECs on hepatocyte proliferation and the corresponding mechanisms.

### Conditioned medium (CM) from VECs with active Piezo1 can promote proliferation and epithelial–mesenchymal transition (EMT) of hepatocytes *in vitro*

To investigate the potential of Piezo1 activation in VECs to induce hepatocyte proliferation, we collected CM from VECs where Piezo1 had been activated by Yoda1 (a specific agonist of Piezo1), HCM^Yoda1^ (CM from HUVEC cells), and SCM^Yoda1^ (CM from SK-Hep1 cells); all promoted the proliferation of hepatocytes (Fig. 2A, B). Interestingly, we observed a concomitant weakening of intercellular connections and the disappearance of colony growth, with a gradual scattering of cells (Fig. 2C; Fig. S3A). We next evaluated the expression of E-cadherin (cadherin 1/CDH1, an epithelial marker) and vimentin (VIM, a mesenchymal marker) in hepatocytes using qRT-PCR; HCM^Yoda1^ and SCM^Yoda1^ significantly down-regulated CDH1 and up-regulated VIM expression (Fig. 2D; Fig. S3B). Taken together, our results indicate that the activation of Piezo1 in VECs contributes to the proliferation and EMT of hepatocytes.

**Fig. 2:**
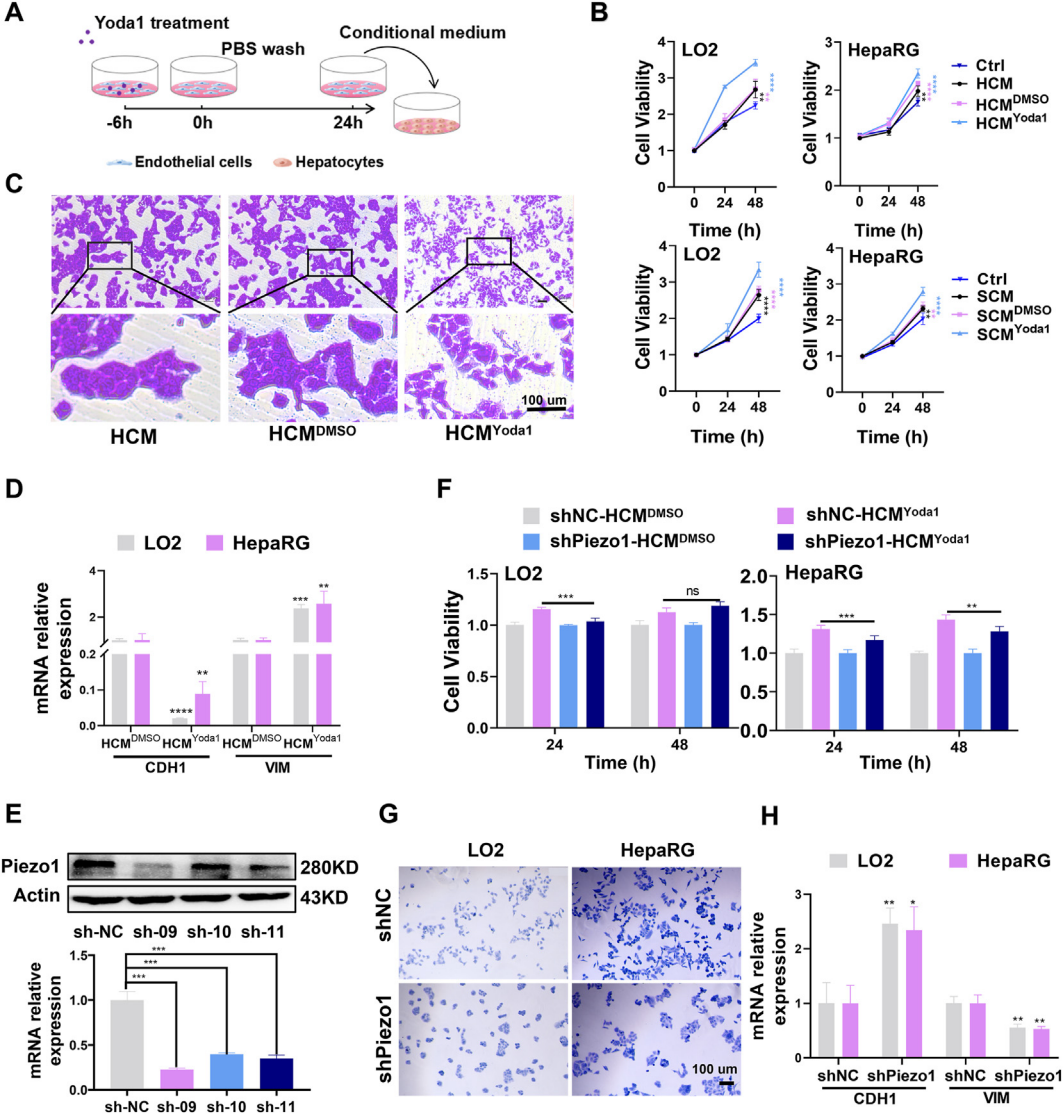
Conditioned medium from VECs with Piezo1 activated by Yoda1 promotes hepatocyte proliferation and epithelial–mesenchymal transition. **(A)** Schematic of endothelial cells treated with Yoda1 and collection of conditioned medium. **(B)** The MTT assay during 48 h revealed that CM^Yoda1^ of VECs could promote the proliferation of hepatocytes (the results of statistical analysis were obtained by comparing with the Ctrl group.). **(C)** The crystal violet staining revealed morphological changes in hepatocytes induced by HCM^Yoda1^. **(D)** HCM^Yoda1^ promotes epithelial–mesenchymal transition in hepatocytes as manifested by changes in the mRNA expression levels of cadherin 1 (CDH1) and vimentin (VIM). **(E)** The WB and qRT-PCR results showed that shRNA knocked down the expression of the Piezo1 gene in HUVEC. **(F–H)**. Knockdown of Piezo1 in VECs can inhibit hepatocyte proliferation (F), morphological changes (G), and epithelial–mesenchymal transition (H) induced by HCM^Yoda1^. ∗*P* < 0.05, ∗∗*P* < 0.01, ∗∗∗*P* < 0.001, ∗∗∗∗*P* < 0.0001; two-tailed Student's *t*-tests. HCM^Yoda1^, conditioned medium VECs with Yoda1-activated Piezo1.

Next, we employed shRNA to knock down the expression of Piezo1 in VECs (Fig. 2E; Fig. S3C, D). The knockdown of Piezo1 resulted in a weakened ability of the CM to promote hepatocyte proliferation (Fig. 2F; Fig. S3E). Additionally, we observed that hepatocyte distribution transformed from scattered to colony growth (Fig. 2G; Fig. S3F). The qRT-PCR analysis showed an increase in CDH1 expression and a decrease in VIM expression levels in hepatocytes (Fig. 2H; Fig. S3G). These results collectively suggest that Piezo1 activation in VECs promotes hepatocyte proliferation and EMT *in vitro*.

### Piezo1 activation promotes the up-regulation of the ERBB and MAPK signaling pathways in VECs

Differential mRNA expression between vehicle- and Yoda1-treated HUVEC and SK-Hep1 cells at 6 h (Fig. S4) revealed the presence of 790 upregulated and 570 downregulated genes in HUVEC and 1472 upregulated and 1022 downregulated genes in SK-Hep1 cells after Yoda1 treatment for 6 h (Fig. 3A). KEGG pathway enrichment analysis revealed that pathways related to MAPK (mitogen-activated protein kinase) and ERBB (the epidermal growth factor family of receptor tyrosine kinases) were enriched in both HUVEC and SK-Hep1 cell lines after Yoda1 treatment (Fig. 3B). The overlapping gene list of up-regulated genes in Yoda1-induced HUVEC and SK-Hep1 cell lines with ligand genes from the online website CellTalkDB were further analyzed using KEGG analysis on the DAVID website. Signaling pathways related to transmembrane receptor protein tyrosine kinase, ERBB, MAPK, and extracellular signal-regulated kinase 1/2 (ERK1/2) were enriched in secretory ligand genes following Yoda1 induction (Fig. 3C). Additionally, gene set enrichment analysis of the sequencing results using KEGG gene sets in GSEA software revealed that ERBB signaling was up-regulated in Yoda1-treated HUVEC (normalized enrichment score = 1.41, nominal *P* < 0.001, false discovery rate = 0.201) and SK-Hep1 (normalized enrichment score = 1.90, nominal *P* < 0.001, false discovery rate = 0.046) cells (Fig. 3D). Notably, 13 genes were co-up-regulated in the Yoda1-induced ERBB signaling pathway in both HUVEC and SK-Hep1 cell lines, with heparin binding epidermal growth factor (HBEGF), EREG, AREG and neuregulin 1 belonging to the secreting ligand gene category (Fig. 3E). This implies that Yoda1-induced changes in gene expression in VECs were prominently observed within the ERBB and MAPK signaling pathways.

**Fig. 3:**
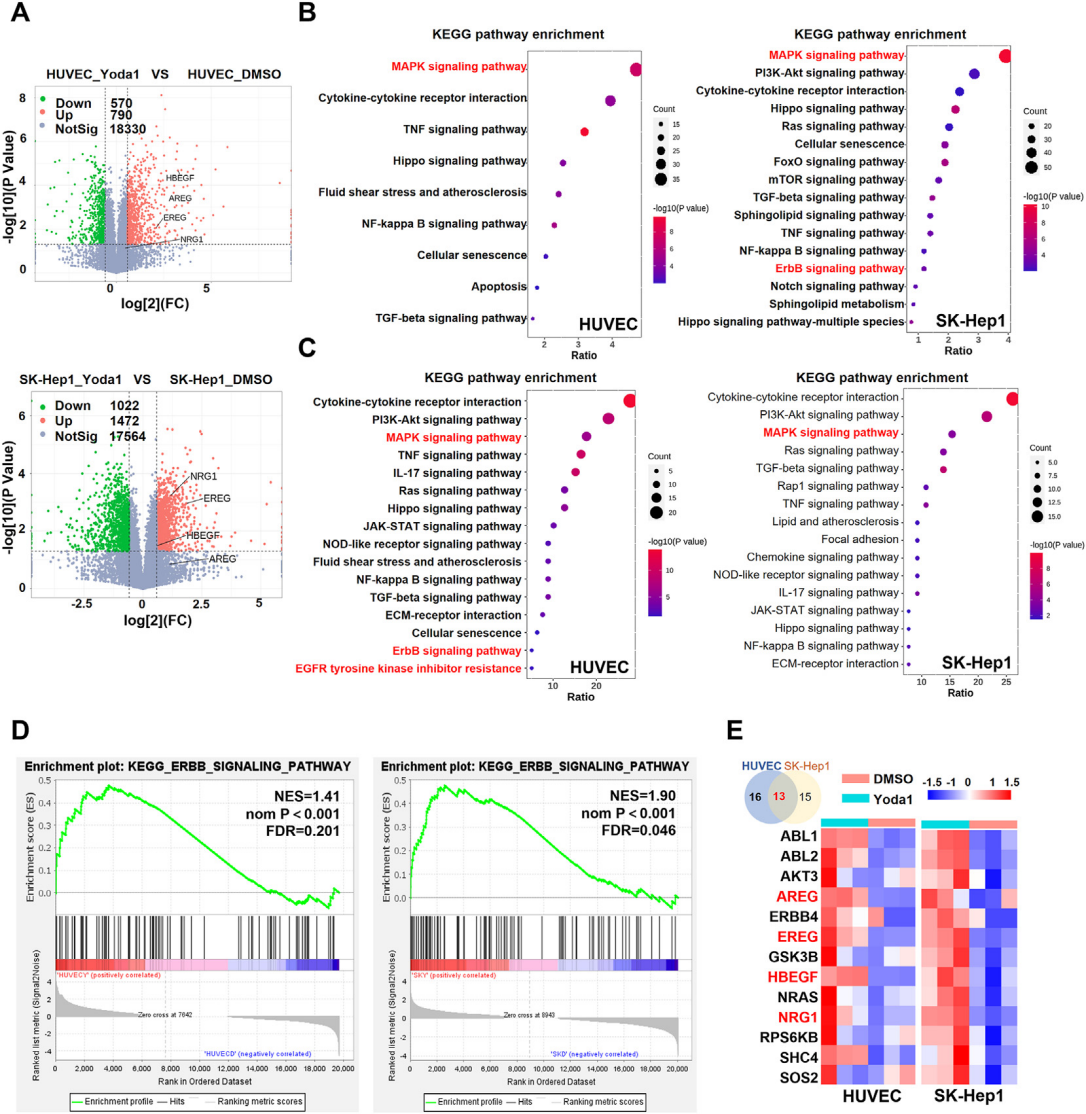
ERBB and MAPK signaling pathway gene expression are up-regulated in vascular endothelial cells (VECs) with Piezo1 activated by Yoda1. **(A)** Volcano plot of differentially expressed genes in HUVEC and SK-Hep1 cells between treated with the Yoda1 and DMSO (FC > 1.5, *P* < 0.05). **(B)** Signal pathway from results of KEGG enrichment analysis based on differential up-regulated genes of HUVEC and SK-Hep1 cells after treatment with Yoda1 *vs*. DMSO (*P* < 0.05). **(C)** Signal pathway obtained by KEGG enrichment analysis based on the overlap of genes in the ligand library and up-regulated genes in HUVEC and SK-Hep1 cell lines (*P* < 0.05). The ligand library is from the CellTalkDB website (http://tcm.zju.edu.cn/celltalkdb/download.php). **(D)** Gene set enrichment analysis suggested that the ERBB signal pathway genes were up-regulated in HUVEC and SK-Hep1 cell lines after Yoda1 activates Piezo1. **(E)** HBEGF, EREG, AREG, and neuregulin 1 (NRG1) expression were elevated in both HUVEC and SK-Hep1 cells with Piezo1 activated by Yoda1.

### PKC and ERK1/2 contribute to regulation of the expression of HBEGF, EREG and AREG in VECs under Piezo1 activation

Mouse primary vascular endothelial cells were isolated and cultured (Fig. S5A), and qRT-PCR results indicated that Yoda1 induced an increase in the mRNA expression of HBEGF, EREG, and AREG in VECs (HUVEC, SK-Hep1, and Mouse primary vascular endothelial cells) (Fig. 4A). Furthermore, Piezo1 knockdown led to an obvious inhibition of the HBEGF, EREG, and AREG expression in VECs treated with Yoda1 (Fig. 4B; Fig. S5B). In HCM^Yoda1^ and SCM^Yoda1^, we found that the levels of AREG and EREG proteins were significantly elevated (Fig. 4C; Fig. S5C), and Piezo1 knockdown led to a noticeable inhibition of EREG and AREG secretion in HUVEC (Fig. 4D). Mechanical forces have been shown to increase HBEGF secretion in various cell types, and its role in promoting hepatocyte proliferation and LR is well established. We investigated whether the MAPK family played a role in Yoda1-induced HBEGF, EREG, and AREG expression in VECs. First, four specific inhibitors with different targets were utilized to examine the regulation of HBEGF, AREG, and EREG by MAPK and downstream signaling pathways, namely ravoxertinib (a selective inhibitor of ERK1/2), adezmapimod SB 203580 (a selective inhibitor of p38 MAPK), SP600125 (a selective inhibitor of c-Jun N-terminal kinase), and SR11302 (a selective inhibitor of activator protein-1, a downstream target of c-Jun N-terminal kinase). The qRT-PCR results showed that ravoxertinib could significantly inhibit the expression of HBEGF, EREG, and AREG, while adezmapimod SB 203580 could only inhibit the expression of AREG and EREG. However, SP600125 and SR11302 had little effect on the expression of the three genes (Fig. 4E). The WB results suggested that Yoda1 induced an increase in ERK1/2 phosphorylation (Fig. S5D), and that knockdown of Piezo1 reduced this increase (Fig. 4F; Fig. S5E).

**Fig. 4:**
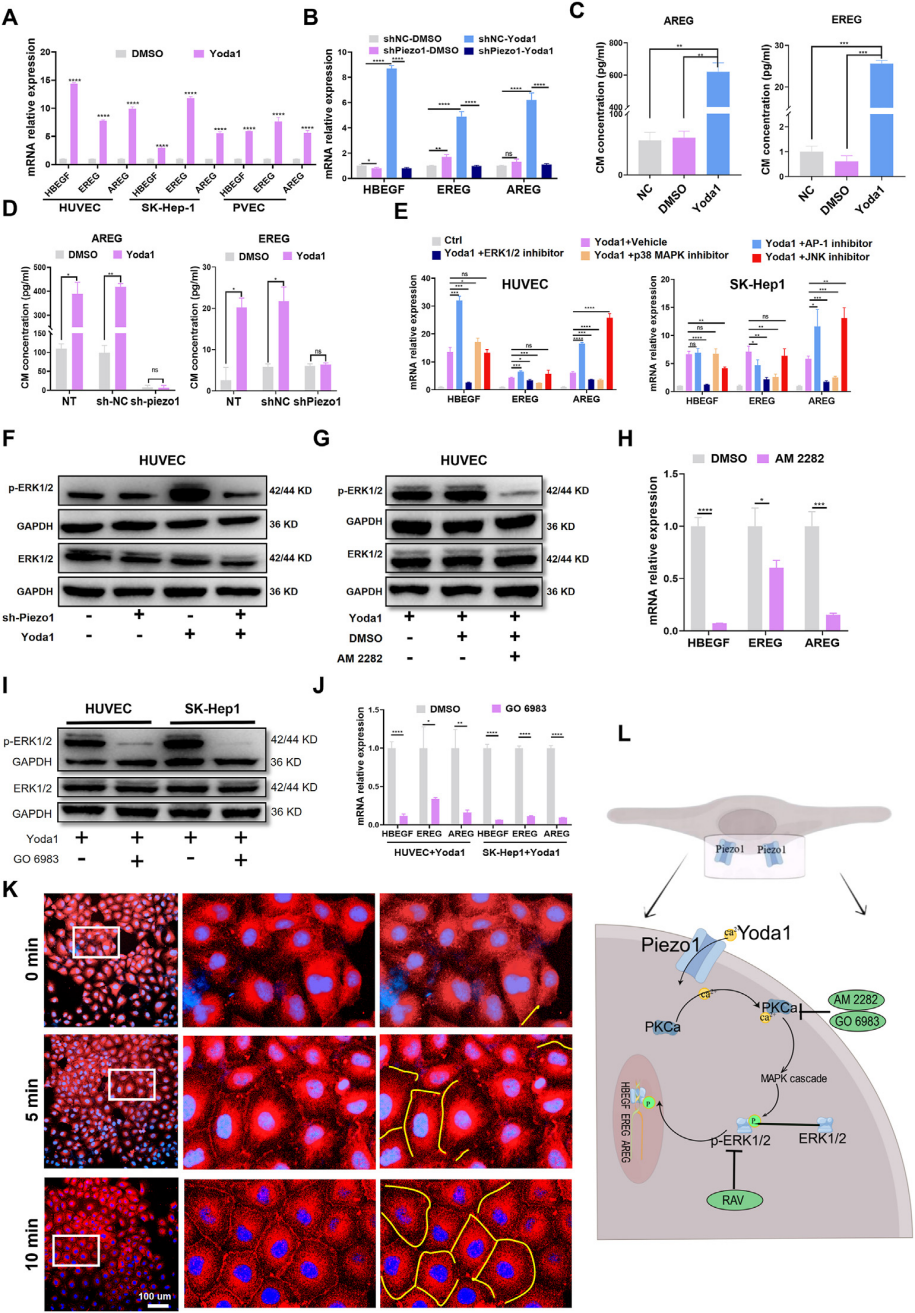
Expression of HBEGF, EREG, and AREG induced by Piezo1 activation in vascular endothelial cells (VECs) depends on PKC/ERK1/2 signaling pathways. **(A, B)** The qRT-PCR results showed increased mRNA expression of HBEGF, EREG, and AREG induced by Yoda1 in endothelial cells and was reversed with Piezo1 knockdown in HUVEC cells. **(C, D)** the ELISA assay results showed that increased secretion of EREG and AREG in HCMYoda1 were reversed with Piezo1 knockdown. **(E)** The qRT-PCR results showed the block effect of ravoxertinib (ERK1/2 inhibitor, 5 μM), adezmapimod SB 203580 (P38 MAPK inhibitor, 10 μM), SP (c-Jun N-terminal kinase inhibitor, 25 μM), and SR11302 (activator protein-1 inhibitor, 10 μM) on the expression of HBEGF, EREG, and AREG induced by Yoda1 in HUVEC and SK-Hep1 cells. **(F)** WB suggests that the knockdown of Piezo1 can inhibit the activation of ERK1/2 induced by Yoda1. **(G, H)** The WB and qRT-PCR results for non-selective protein kinase inhibitor AM2282 (200 nM) effect on activation of ERK1/2 and the expression of HBEGF, AREG, and EREG in HUVEC cells induced by Yoda1. **(I, J)** WB and qRT-PCR confirmed that selective protein kinase C inhibitor GO 6983 (10 μM) could inhibit the activation of ERK1/2 and the expression of HBEGF, AREG, and EREG in endothelial cells induced by Yoda1. **(K)** Immunofluorescence analysis revealed that Yoda1 (5 μM for 0, 5, and 10 min) could activate PKCα. **(L)** The schematic diagram delineating the probable mechanism of the expression of HBEGF, EREG, and AREG induced by Piezo1 activated by Yoda1. ∗*P* < 0.05, ∗∗*P* < 0.01, ∗∗∗*P* < 0.001, ∗∗∗∗*P* < 0.0001; two-tailed Student's *t*-tests. PVEC, mouse primary vascular endothelial cell; HCMYoda1, conditioned medium from HUVEC with Yoda1-activated Piezo1.

Piezo1 activation can activate multiple protein kinases such as protein kinase A, protein kinase B, and protein kinase C (PKC). To determine which protein kinase mediates the enhanced phosphorylation of ERK1/2, we conducted a series of experiments. The nonselective protein kinase inhibitor AM 2282 was used to block the activation of protein kinase A and PKC, which significantly inhibited ERK1/2 activation and the expression of HBEGF, AREG, and EREG induced by Yoda1 in VECs (Fig. 4G, H; Fig. S5F, G). Moreover, the PKC-specific inhibitor GO 6983 also blocked Yoda1-induced ERK1/2 activation and the expression of HBEGF, AREG, and EREG (Fig. 4I, J). To further confirm the role of PKC in the biological effects induced by Yoda1, we used phorbol 12-myristate 13-acetate, an established PKC agonist, to promote ERK1/2 phosphorylation and elevated expression of HBEGF, EREG, and AREG in VECs (Fig. S6A–D). These results suggest that the activation of PKC and ERK1/2 play a crucial role in the enhanced expression levels of HBEGF, AREG and EREG resulting from Yoda1-induced Piezo1 activation.

To further elucidate the relationship between Yoda1-induced Piezo1 activation and PKC activation, we performed immunofluorescence staining to examine the changes in PKCα, a critical member of the classical PKC family. First, we confirmed that Piezo1 was involved in regulating the expression of PKCα. The qRT-PCR and WB results demonstrated that Piezo1 knockdown did not affect the mRNA and protein expression of PKCα in HUEVC and SK-Hep1 cells (Fig. S6E). Membrane translocation is a vital feature of PKCα activation. Yoda1 rapidly induced the membrane translocation of PKCα in both HUEVC and SK-Hep1 cells (Fig. 4K; Fig. S6F). In summary, our results indicate that PKCα and ERK1/2 participate in regulating the expression of HBEGF, EREG and AREG in VECs where Piezo1 is activated by Yoda1 (Fig. 4L).

Given the potential for Piezo1 activation to trigger a calcium ion influx and subsequently initiate a biological cascade, we embarked on an investigation into the expression of HBEGF, EREG, and AREG in endothelial cells following Yoda1 stimulation in the absence of extracellular calcium ions. Our findings indicated that the elimination of extracellular calcium ions, facilitated by EDTA and a calcium-free medium, significantly reduced the Yoda1-induced expression of EREG and AREG (Fig. S7A). To gauge intracellular calcium concentrations, we employed the Fluo-4AM calcium detection kit. The outcomes demonstrated that Yoda1 was capable of swiftly elevating intracellular calcium levels (Fig. S7B, C).

### Hepatocyte proliferation and partial EMT induced by AREG and EREG depend on EGFR

Based on RNA sequencing data from mouse liver tissue in the GEO database (GSE169242), the expression level of EGFR is the highest in the ERBB family (Fig. S8A). We thus hypothesized that CM from VECs with Yoda1-activated Piezo1 (CM^Yoda1^) could activate EGFR and promote the proliferation and EMT of hepatocytes. The WB results revealed that CM^Yoda1^ significantly increased the phosphorylation levels of EGFR in LO2 and HepaRG cells (Fig. 5A). The promoting effect of CM^Yoda1^ on the proliferation of LO2 and HepaRG cells was blocked by gefitinib, an EGFR-specific inhibitor (Fig. 5B). Morphological observations indicated that the morphology of hepatocytes was restored by gefitinib (Fig. 5C; Fig. S8B). The qRT-PCR results showed that gefitinib partially reversed the mRNA expression level of CDH1 but had no significant effect on the expression of VIM (Fig. 5D; Fig. S8C). These results suggest that CM^Yoda1^ could promote the proliferation and partial EMT of hepatocytes through the EGFR signaling pathway, which is characterized by the loss of epithelial characteristics with little effect on the expression of mesenchymal components.

**Fig. 5:**
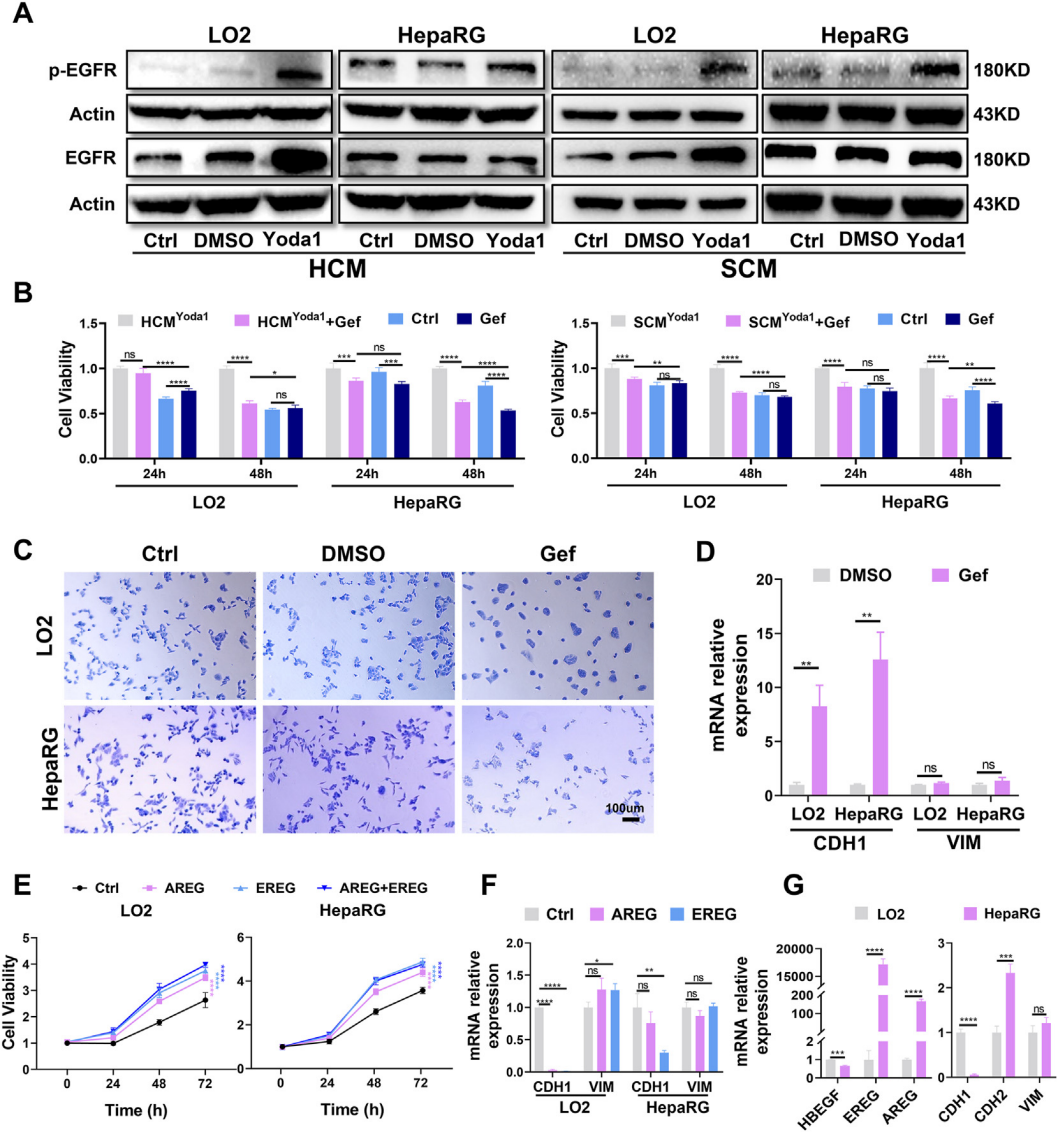
AREG and EREG from vascular endothelial cells (VECs) promote hepatocyte proliferation and partial epithelial–mesenchymal transition through the EGFR signal pathway. **(A)** WB revealed that EGFR was activated following treatment with HCMYoda1 and SCMYoda1. **(B)** The MTT assay during 48 h proved that gefitinib could block the proliferation of hepatocytes induced by HCMYoda1 and SCMYoda1. **(C)** The crystal violet staining demonstrated that the morphological changes of hepatocytes induced by HCMYoda1 were inhibited by gefitinib. **(D)** qRT-PCR analysis suggested that Gefitinib reversed the reduction of cadherin 1 (CDH1) induced by HCMYoda1 but had no significant effect on vimentin (VIM). **(E)** The MTT assay during 72 h confirmed that the recombinant proteins of AREG and EREG could promote the proliferation of hepatocytes (the results of statistical analysis were obtained by comparing with the Ctrl group). **(F)** qRT-PCR analysis proved that AREG and EREG promoted partial epithelial–mesenchymal transition of hepatocytes. **(G)** Differences in basal expression levels of HBEGF, AREG, EREG, CDH1, cadherin 2 (CDH2), and VIM between LO2 and HepaRG cells. ∗*P* < 0.05, ∗∗*P* < 0.01, ∗∗∗*P* < 0.001, ∗∗∗∗*P* < 0.0001; two-tailed Student's *t*-tests. HCMYoda1, conditioned medium from HUVEC with Yoda1-activated Piezo1; SCMYoda1, conditioned medium from SK-Hep1 with Yoda1-activated Piezo1.

Both AREG and EREG showed similar results to HCM^Yoda1^ and SCM^Yoda1^ in terms of hepatocyte morphological changes and proliferation (Fig. 5E; Fig. S8D). The qRT-PCR results indicated that AREG and EREG significantly decreased CDH1 expression in LO2 cells and had little effect on VIM; both had relatively minor effects on CDH1 and VIM expression in HepaRG cells (Fig. 5F). The observed differences in the response of LO2 and HepaRG cells to AREG and EREG likely reflect differences in the affinity of AREG and EREG for EGFR and in the basal state of the two cell lines. The qRT-PCR results indicated mRNA expression levels of CDH1 in HepaRG cells were significantly lower than that in LO2 cells. In addition, the levels of AREG and EREG were approximately 160 and 18,000 times higher, respectively, in HepaRG cells than in LO2 cells (Fig. 5G).

### AREG and EREG enhance both proliferation and EMT in mouse primary hepatocytes cultured *in vitro*

To further elucidate the impact of VEC-derived AREG and EREG on hepatocyte proliferation, mouse primary hepatocytes were isolated for subsequent experimentation (Fig. S9). The MTT assay outcomes indicated that the recombinant proteins AREG and EREG significantly enhanced the proliferation of mouse primary hepatocytes (Fig. 6A). Additionally, EdU assays corroborated an acceleration in cell proliferation, accompanied by an up-regulation of Ki67 and CyclinD1 protein expression (Fig. 6B–F). EMT plays a critical role in the LR process. Following the administration of recombinant proteins AREG and EREG, a notable transformation in the morphology of mouse primary hepatocytes was observed at 72 h, with cells adopting a fusiform shape (Fig. 6G). qRT-PCR and WB analyses revealed a down-regulation of CDH1 (E-cadherin) expression and an up-regulation of VIM expression after AREG and EREG treatment (Fig. 6H, I). These findings collectively indicate that AREG and EREG could foster the proliferation and EMT of mouse primary hepatocytes *in vitro*.

**Fig. 6:**
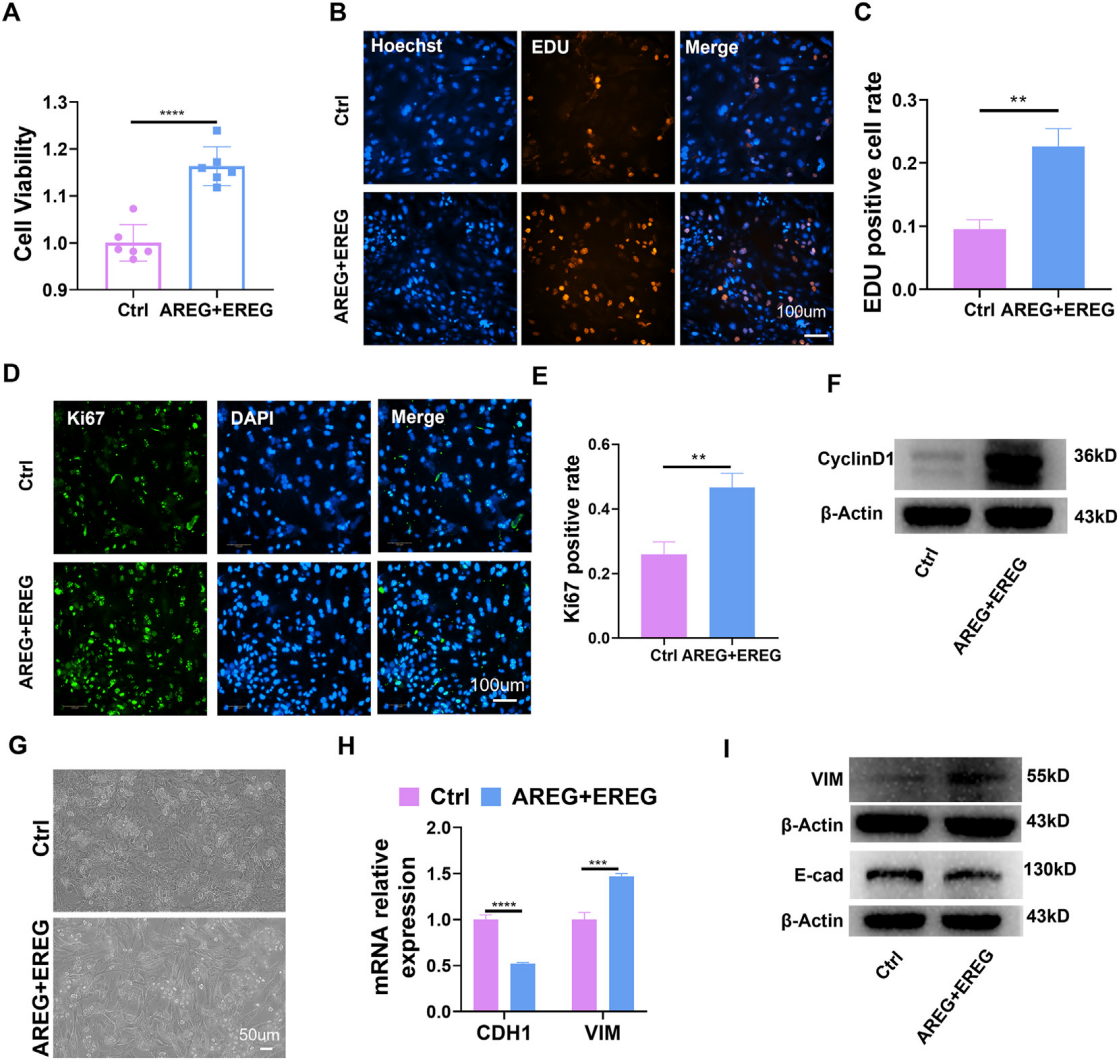
AREG and EREG promote the proliferation and epithelial–mesenchymal transition of mouse primary hepatocytes *in vitro*. **(A)** MTT assay showed that AREG and EREG promoted the proliferation of mouse primary hepatocytes. **(B, C)** EDU detected that AREG and EREG promoted the proliferation of mouse primary hepatocytes. **(D, E)** Immunofluorescence assay showed that AREG and EREG promoted the expression of Ki67 in mouse primary hepatocytes. **(F)** WB detected that AREG and EREG promoted the expression of CyclinD1 in mouse primary hepatocytes. **(G)** AREG and EREG promoted the morphological changes of mouse primary hepatocytes. **(H)** The results of qRT-PCR showed that AREG and EREG promoted the down-regulation of cadherin 1 (CDH1) expression and up-regulation of vimentin (VIM) expression in mouse primary hepatocytes. **(I)** WB showed that AREG and EREG promoted the decrease of E-cadherin expression and the increase of VIM expression. ∗*P* < 0.05, ∗∗*P* < 0.01, ∗∗∗*P* < 0.001, ∗∗∗∗*P* < 0.0001; two-tailed Student's *t*-tests. E-cad, E-cadherin.

### EGFR mediates hepatocyte proliferation *in vitro* and LR *in vivo*

In light of the distinct baseline states of the two hepatocyte cell lines under investigation, we aimed to focus our inquiry on LO2 cells to explore the mechanisms of AREG and EREG on the promotion of hepatocyte proliferation and partial EMT. Both AREG and EREG enhanced the phosphorylation of EGFR in LO2 cells (Fig. 7A), and proliferative and partial EMT effects were abrogated by gefitinib treatment, which also restored morphological changes (Fig. 7B; Fig. S10A). Our results suggest that AREG and EREG's roles in promoting LO2 cell proliferation and partial EMT are, to some extent, dependent on EGFR. With ERK1/2 serving as an important mediator of the ERBB signaling pathway (Fig. 7C), ravoxertinib could partially block the promoting effects of AREG and EREG on LO2 cell proliferation (Fig. 7D), while qRT-PCR assays revealed that ravoxertinib had a significant effect on the recovery of CDH1 (Fig. S10B). The effects of AREG and EREG on p-ERK1/2 were not blocked by gefitinib (Fig. 7E), indicating the effect of AREG and EREG on LO2 cells is mediated through the EGFR and ERK1/2 signaling pathways, respectively (Fig. 7F). Notably, EGFR activation primarily mediated LO2 cell proliferation while ERK1/2 activation mediated partial EMT.

**Fig. 7:**
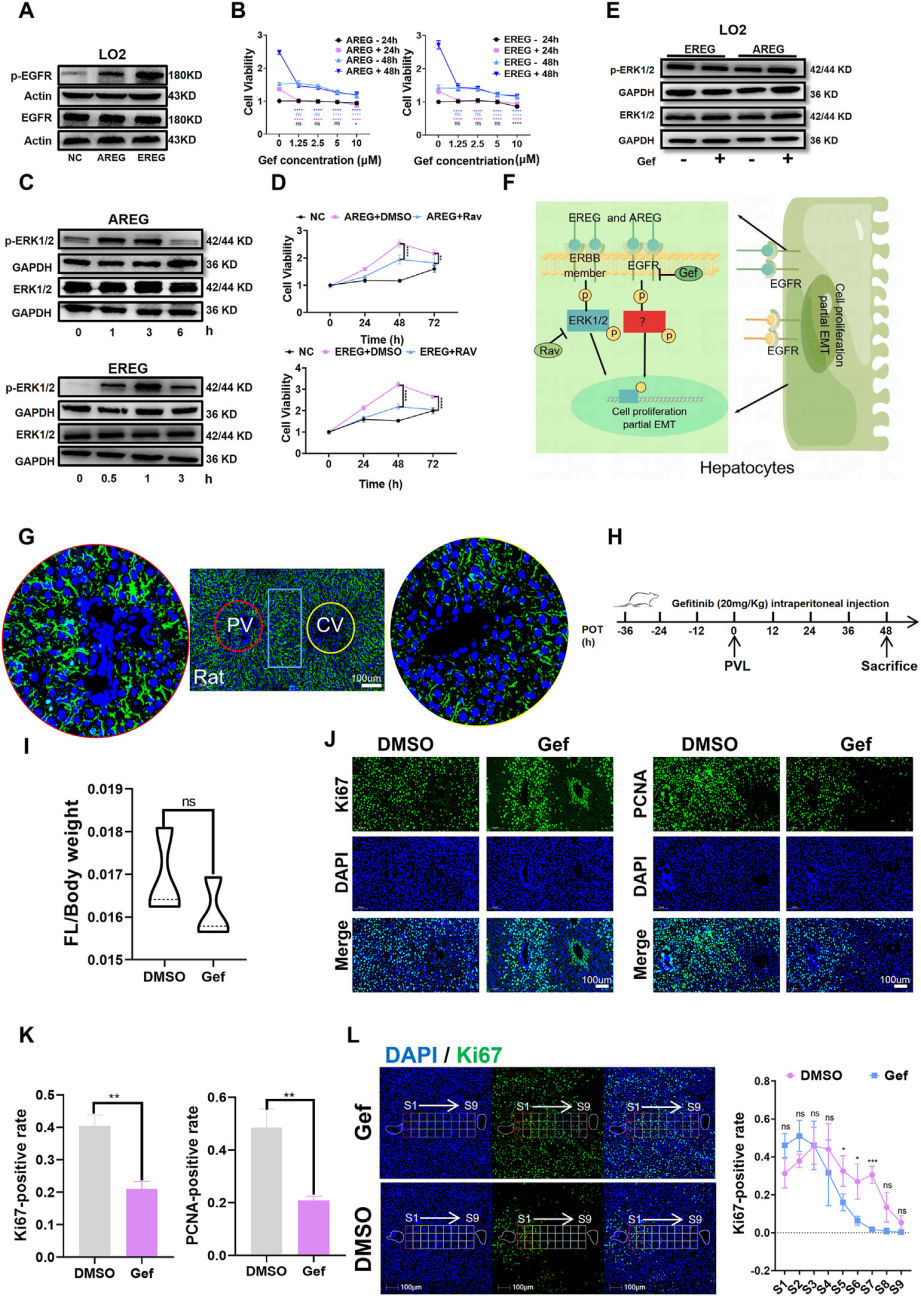
EGFR mediates hepatocyte proliferation and liver regeneration. **(A)** WB revealed that EGFR was activated by recombinant proteins of AREG (50 ng/mL) and EREG (20 ng/mL) in LO2 cells. **(B)** The MTT assay at 24 and 48 h suggested that gefitinib could block the proliferation of hepatocytes induced by AREG (50 ng/mL) and EREG (20 ng/mL) in LO2 cells (the results of statistical analysis were obtained by comparing with the 0 μM Gef group, respectively). **(C)** WB demonstrated that ERK1/2 was activated by recombinant proteins of AREG (50 ng/mL) and EREG (20 ng/mL) in LO2 cells. **(D)** The MTT assay confirmed that the proliferation of LO2 cells induced by AREG and EREG was partially reversed by ravoxertinib (ERK1/2 inhibitor) (the results of statistical analysis were obtained by comparing with the AREG+DMSO group). **(E)** WB indicated that activation of ERK1/2 induced by recombinant proteins of AREG and EREG were not inhibited by gefitinib. **(F)** Schematic diagram of the proposed mechanism by which AREG and EREG promote hepatocyte proliferation and partial epithelial–mesenchymal transition. **(G)** Immunofluorescence analysis revealed higher expression of EGFR in hepatocytes of rat tissues in zone 1 and zone 2 than in zone 3. **(H)** Schematic diagram of the application scheme of gefitinib and vehicle. **(I)** The future liver weight (FLW) to body weight (BW) ratio was calculated after PVL 48 h in the vehicle and gefitinib groups. **(J, K)** Immunofluorescence staining of Ki67 and PCNA in liver sections from DMSO (*n* = 3) and gefitinib (*n* = 3) rats at 48 h after PVL. Ki67-and PCNA-positive hepatocytes were counted in three PV-CV fields, and the average count of these three fields was set as the Ki67-and PCNA-positive cell number of one rat. **(L)** Gefitinib inhibited the proliferation of S5–S7 hepatocytes at 48 h after PVL and delayed liver regeneration. ∗*P* < 0.05, ∗∗*P* < 0.01, ∗∗∗*P* < 0.001, ∗∗∗∗*P* < 0.0001; two-tailed Student's *t*-tests.

To investigate the potential role of EGFR in the distribution of proliferating hepatocytes during the early stages of PVL-induced LR, we first examined the expression of EGFR in human liver tissue using an online database (https://www.proteinatlas.org) and found that its expression was relatively higher in hepatocytes in zone 1 and zone 2, and relatively lower in zone 3 (Fig. S11). To further explore the distribution of EGFR expression in hepatic lobules, we performed immunofluorescence staining on rat liver tissue sections. EGFR expression was higher in zone 1 and zone 2 hepatocytes and relatively lower in zone 3 hepatocytes (Fig. 7G). This “non-central venous periphery” distribution of EGFR is similar to the distribution of Ki67-positive hepatocytes in the regenerating liver induced by PVL at 24–48 h. To investigate whether EGFR mediates the regional distribution of proliferating cells in the liver during the early stage of regeneration induced by PVL, we used gefitinib to inhibit EGFR activity (Fig. 7H). The drug and vehicle were administered intraperitoneally every 12 h starting 36 h before PVL and continuing until 48 h after PVL in the gefitinib and control groups. Gefitinib inhibited PVL-induced hepatocyte proliferation, as evidenced by the reduced positive rates of Ki67 and PCNA in one regenerating unit (within the range of portal vein–central vein) in the gefitinib group (Fig. 7I–K). To investigate whether gefitinib influenced regional LR, hepatocytes from the portal vein to central vein areas were categorized into nine distinct layers, and the Ki67 positivity rate was compared across these layers. Our findings revealed that the application of gefitinib primarily suppressed the proliferation of hepatocytes in layers 5–7 of the regenerating liver at 48 h after PVL (Fig. 7L). Taken together, these findings suggest that activation of EGFR may mediate PVL-induced proliferation of hepatocytes during LR and that gefitinib delays the process of LR.

### Yoda1 enhances LR impaired by fasting *in vivo*

The portal vein, accounting for approximately 70% of the liver's blood supply, transports blood from the digestive tract to the liver, carrying most nutrients. Fasting can impact blood flow and nutrient delivery, potentially interfering with LR. We conducted a series of experiments to determine Yoda1's efficacy in ameliorating impaired LR. Initially, postoperative fasting resulted in a decreased blood flow velocity in the portal vein of the regenerating liver following PVL (Fig. 8A, B). Immunofluorescence was utilized to assess Ki67 expression in the regenerated liver tissues of two rat groups. The findings revealed a significant suppression of Ki67 expression in the fasting group's regenerated liver tissues (Fig. 8C, D). Fasting precipitated a rapid reduction in total liver weight in rats, with no notable difference in the proportion of liver lobes (Fig. S12A, B). Is the diminished Ki67 expression in the regenerative liver of the fasting group attributable to inadequate mechanical stimulation and Piezo1 activation of VECs due to reduced portal vein blood flow? To explore whether exogenous activation of Piezo1 could expedite LR, we administered Yoda1 intraperitoneally to fasting rats (1 mg/kg body weight, every 12 h starting 36 h before PVL and continuing until 24 h after PVL; Fig. S12C). The outcomes demonstrated that Yoda1 administration led to an increase in Ki67 expression in the regenerated liver tissues, thereby enhancing the LR process (Fig. 8E, F).

**Fig. 8:**
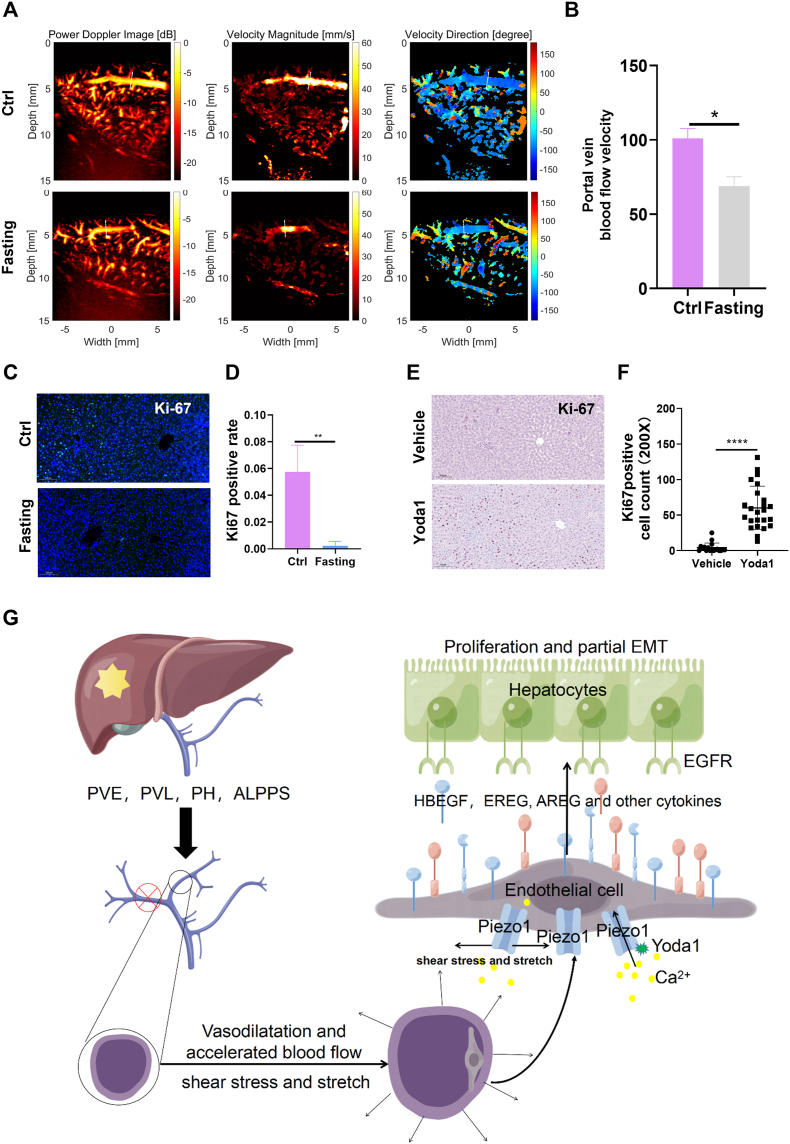
Yoda1 improves fasting-induced poor liver regeneration *in vivo*. **(A)** Ultrasound was used to evaluate the portal vein blood flow velocity in the control and the fasting group (18 h after PVL, *n* = 2). **(B)** Fasting induced a decrease in the velocity of portal vein blood flow in rats. **(C, D)** Ki67 staining was used to evaluate liver regeneration in the control and fasting group at 24 h after PVL (*n* = 3). **(E, F)** Under fasting conditions, Yoda1 promoted the expression of Ki67 in the regenerated liver (six different PV-CV ranges were selected for each rat right liver section to calculate the number of Ki67 positive cells. Vehicle group: *n* = 3; Yoda1 group: *n* = 4; Yoda1: 1 mg/kg). **(G)** Schematic of the mechanism by which piezo1 activation mediates endothelial and hepatocyte crosstalk during liver regeneration. After PVE, PVL, partial hepatectomy (PH), and ALPPS, the change of portal vein blood flow causes the imbalance of intrahepatic mechanical homeostasis. The activation of Piezo1 in endothelial cells can promote the expression of HBEGF, EREG, and AREG, and then activate the EGFR of hepatocytes, thus promoting the proliferation of hepatocytes and EMT. ∗*P* < 0.05, ∗∗*P* < 0.01, ∗∗∗*P* < 0.001, ∗∗∗∗*P* < 0.0001; two-tailed Student's *t*-tests. ALPPS, associated liver partition and portal vein ligation for staged hepatectomy.

## Discussion

Hemodynamic changes are a common feature of liver surgery, and VECs are most closely connected to blood flow in the liver.^15^ Hemodynamic changes induced by surgery inevitably cause significant functional change in VECs, including liver sinusoidal endothelial cells. Previous research has demonstrated the involvement of liver sinusoidal endothelial cells in modulating the initiation and cessation of LR and in regulating the proliferation of hepatocytes during the regeneration process.^8^ Extreme partial hepatectomy-induced liver cell damage can induce postoperative LR disorders and liver failure,^2^^,^^16^ which are important factors limiting liver surgery. However, controlled portal vein blood flow at 3.2 times higher than the baseline can promote hepatocyte proliferation and reduce hepatocyte necrosis.^17^ Therefore, moderate hemodynamic changes can activate mechanosensing structures in VECs, leading to the production of cytokines, which play a crucial role in promoting LR.^6^^,^^8^^,^^9^

The present study demonstrates that activation of Piezo1 in VECs promotes hepatocyte proliferation through the release of HBEGF, EREG, and AREG. The regulatory mechanisms of these growth factors vary across different cell types and stimuli. Previous studies suggest that activator protein-1 is involved in the increased expression of HBEGF induced by tension.^18^ However, our findings indicate that the expression of HBEGF in VECs induced by Yoda1 is not blocked by specific inhibitors of c-Jun N-terminal kinase and activator protein-1, suggesting that activator protein-1 does not mediate the increase of HBEGF expression in this context. Instead, we observed that Yoda1-induced EREG and AREG expression was blocked by inhibitors of the ERK1/2 and P38 MAPK pathways, while ravoxertinib and adezmapimod SB 203580 had different effects on HBEGF expression. Literature suggests that Piezo1 activation induces calcium influx,^19^ leading to the activation of PKC and ERK1/2. PKC inhibitors were able to block Yoda1-induced increases in ERK1/2 phosphorylation and expression levels of HBEGF, EREG, and AREG, indicating that the expression of these growth factors in VECs was regulated by the Piezo1/PKC/ERK1/2 axis.

Blood flow changes can stimulate the expression of various growth and inflammatory factors from VECs, among which hepatocyte growth factor and interleukin-6 are the more common cytokines regulating LR.^9^^,^^20^ HBEGF plays a crucial role in LR and hepatocyte proliferation.^21^ The transcriptional expression of HBEGF rapidly rises at 1.5 h after partial hepatectomy, peaks at 6 h, and continues to increase for up to 72 h. Notably, the expression of HBEGF is primarily observed in non-parenchymal cells, namely Kupffer cells and liver sinusoidal endothelial cells, rather than hepatocytes.^21^ Although overexpression of HBEGF does not affect baseline liver size, it accelerates the restoration of liver weight during LR following partial hepatectomy.^22^ The expression level of AREG rapidly increases after partial hepatectomy in rats, promoting hepatocyte proliferation and DNA synthesis through the ERK1/2 and AKT signaling pathways.^23^ In liver transplantation models, the induction of AREG expression significantly increases with 50% liver volume transplantation but is not significantly increased with 30% liver volume transplantation, resulting in disrupted LR.^24^ Despite with greater mechanical stimulation in the 30% liver transplantation model, AREG expression is significantly inhibited compared with 50% liver transplantation model. The conflicting literature results indicate that the regulation of AREG expression by mechanical stimulation is complex. In addition to being affected by blood flow, cholestatic liver injury, such as primary biliary cirrhosis and primary sclerosing cholangitis, increase expression levels of AREG to protect liver cells from damage.^25^ The expression level of EREG increases rapidly after partial hepatectomy, but the abnormal expression of EREG does not affect the process of LR.^26^ *In vitro* studies have shown that EREG can promote the proliferation of primary hepatocytes and hepatic progenitor cells.^25^ Liver sinusoidal endothelial cells are one of the main sources of HBEGF during regeneration.^21^ Mechanical stretch can stimulate the transcriptional level of AREG in epidermal stem cells,^27^ but whether the activation of Piezo1 in VECs can promote the expression of AREG is still unclear. In this study, we reveal for the first time that activation of Piezo1 in VECs regulates the expression of HBEGF, EREG, and AREG, and that this is the mechanism by which EREG and AREG promote hepatocyte proliferation and partial EMT. These results provide a theoretical basis for further understanding and exploration of the role of VECs in promoting hepatocyte proliferation and LR under mechanical stimulation.

In a mouse model of partial hepatectomy, zone 2 hepatocytes displayed initial proliferation, which gradually spread toward zone 1 and zone 3 hepatocytes. Notably, activation of the EGFR signaling pathway was critical for hepatocyte proliferation.^28^ Despite its high expression level in the ERBB family, the distribution of EGFR remains poorly understood. Our *in vitro* study demonstrated that Piezo1 activation promotes hepatocyte proliferation by secreting EGFR ligands. Could changes in mechanical stimulation of VECs after PVL regulate the distribution of proliferating hepatocytes during the regeneration process? The distribution of EGFR was similar to that of Ki67-positive cells in the early stages of LR. *In vivo* experiments revealed that EGFR inhibition significantly reduces Ki67-and PCNA-positive staining in hepatocytes in middle zone, impairing LR. Our *in vitro* experiments suggest that mechanical stimulation may regulate the regional distribution of hepatocyte proliferation during regeneration through Piezo1-mediated secretion of EGFR ligands from VECs.

Several models exist on the impact of changes in blood flow on LR, including PVL, partial hepatectomy, associated liver partition and portal vein ligation for staged hepatectomy, small-volume liver transplantation, and partial liver transplantation. However, few studies have investigated the distribution of hepatocyte proliferation during regeneration. Our study found that activating Piezo1 in VECs up-regulated the expression of EREG and AREG, which mediated hepatocyte proliferation through EGFR (Fig. 8G). *In vivo* findings suggest that the regional distribution of EGFR may mediate the spatial distribution of hepatocyte proliferation during the early stages of regeneration. This study is the first to link spatial changes in proliferating hepatocytes during LR with the mechanical stimulation-endothelial cell system.

In conclusion, we investigated the effects and mechanisms of Piezo1 activation in VECs on hepatocyte proliferation for the first time. Our results indicate that Piezo1 activation in VECs leads to the up-regulation of HBEGF, EREG, and AREG gene expression in the ERBB signaling pathway through the PKCα-ERK1/2 axis. This, in turn, activates EGFR in hepatocytes, promoting cell proliferation. Our *in vivo* and *in vitro* findings suggest that cytokines secreted by mechanically stimulated VECs partially influence the distribution of hepatocyte proliferation during LR. These results provide partial molecular evidence on the hemodynamically-induced regulation of LR by VECs and offer a theoretical basis for developing more effective methods to promote LR by reducing mechanical damage, such as reducing portal vein blood flow and pressure.

## Funding

This work was supported by the National Key Research and Development Program of China (No. 2022YFA1103400), the CAMS Innovation Fund for Medical Sciences (No. 2019-I2M-5–056), the 10.13039/501100001809National Natural Science Foundation of China (No. 81930119, 92168207, 32371477, 82090051), the Precision Medicine Research Program of 10.13039/501100004147Tsinghua University (No. 10001020612), and the National High Level Hospital Clinical Research Funding (China) (No. 2022-NHLHCRF-LX-03-0102).

## Author contributions

Conceptualization: Yuelei Hu, Guifang Du, Chao Li, Juan Liu, Yunfang Wang, and Jiahong Dong; experiment and design, collection and assembly of data: Yuelei Hu, Guifang Du, and Chao Li; article writing and revise: Yuelei Hu, Guifang Du, and Juan Liu; collection of ultrasound localization microscopy data and analyses: Yuelei Hu and Rui Wang; review and editing of article: Juan Liu, Yunfang Wang, and Jiahong Dong.

## Conflict of interests

The authors declared no conflict of interests.

## Figures and Tables

**figs1:**
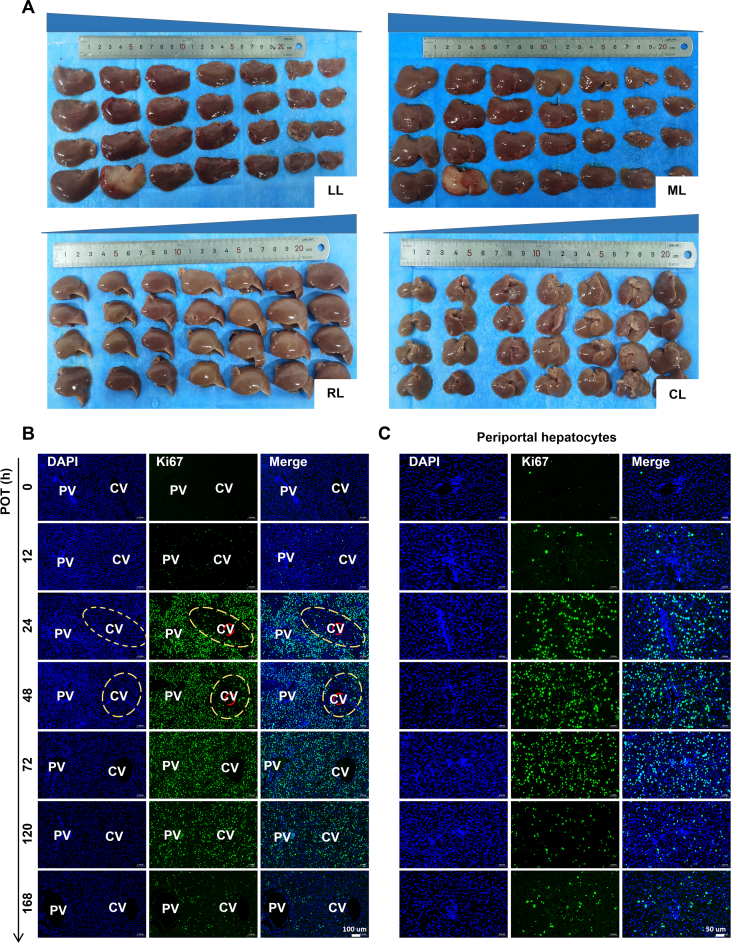


**figs2:**
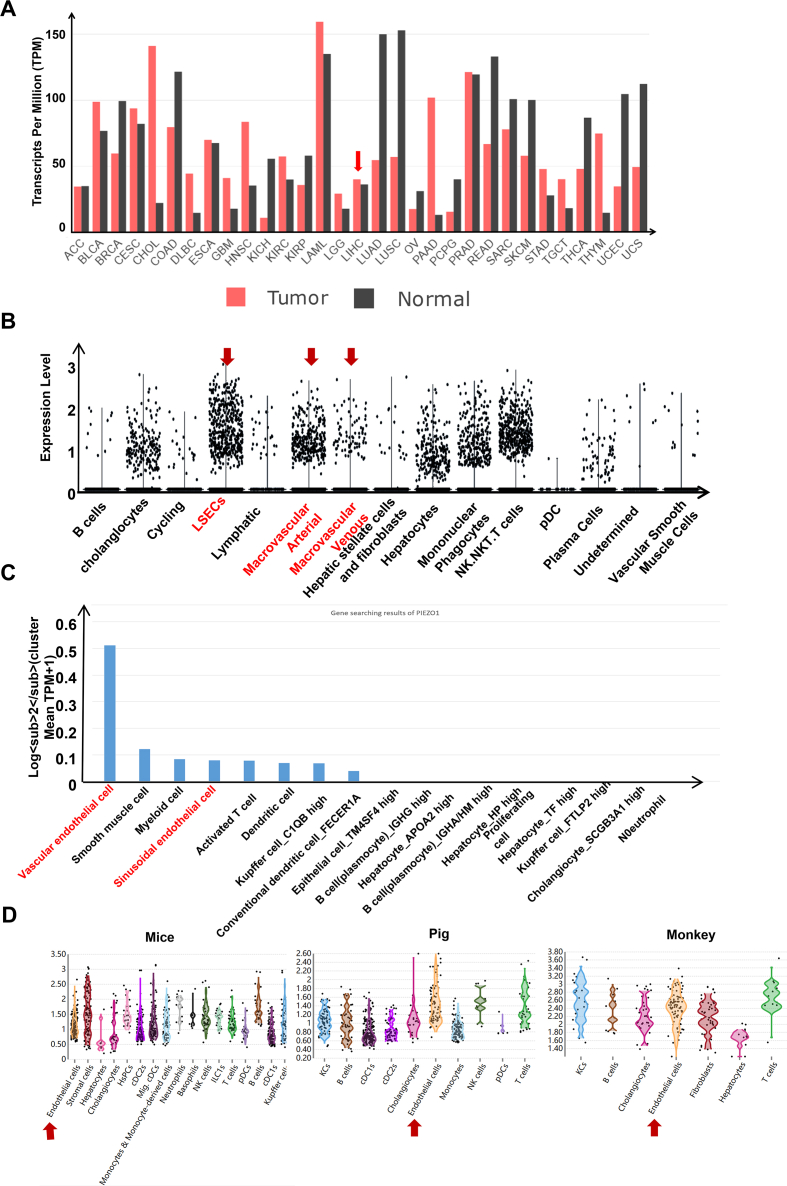


**figs3:**
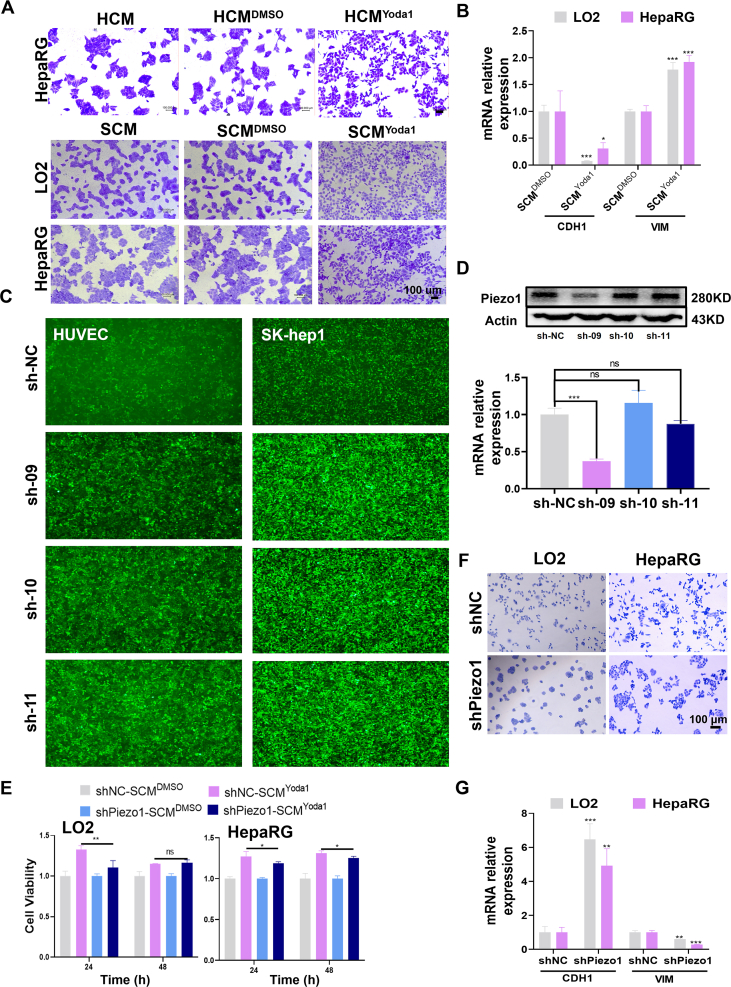


**figs4:**
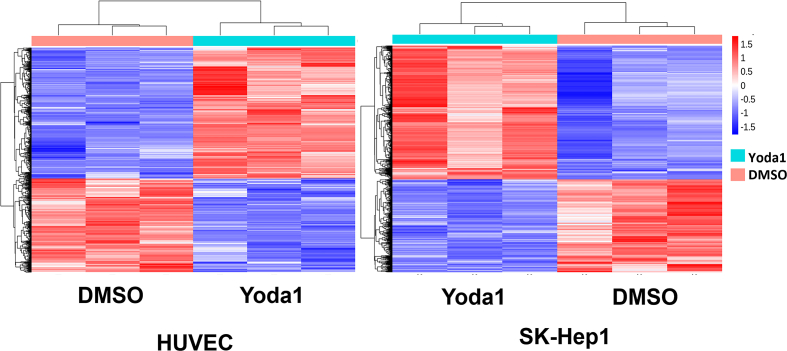


**figs5:**
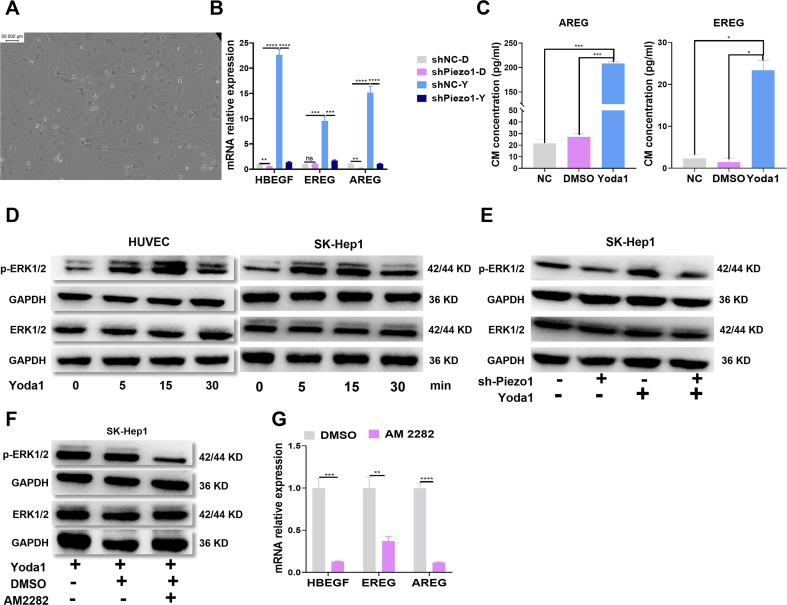


**figs6:**
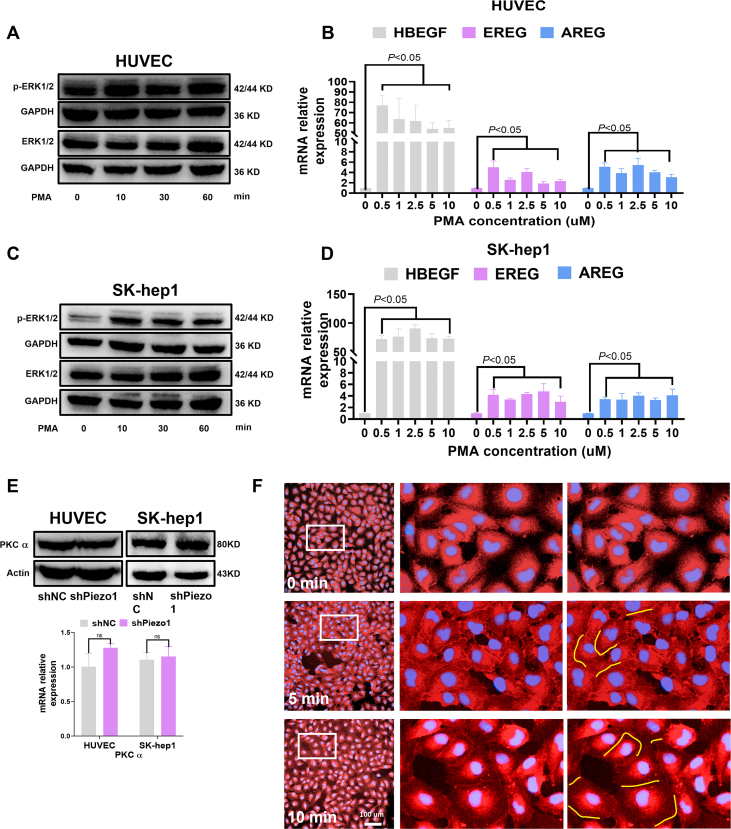


**figs7:**
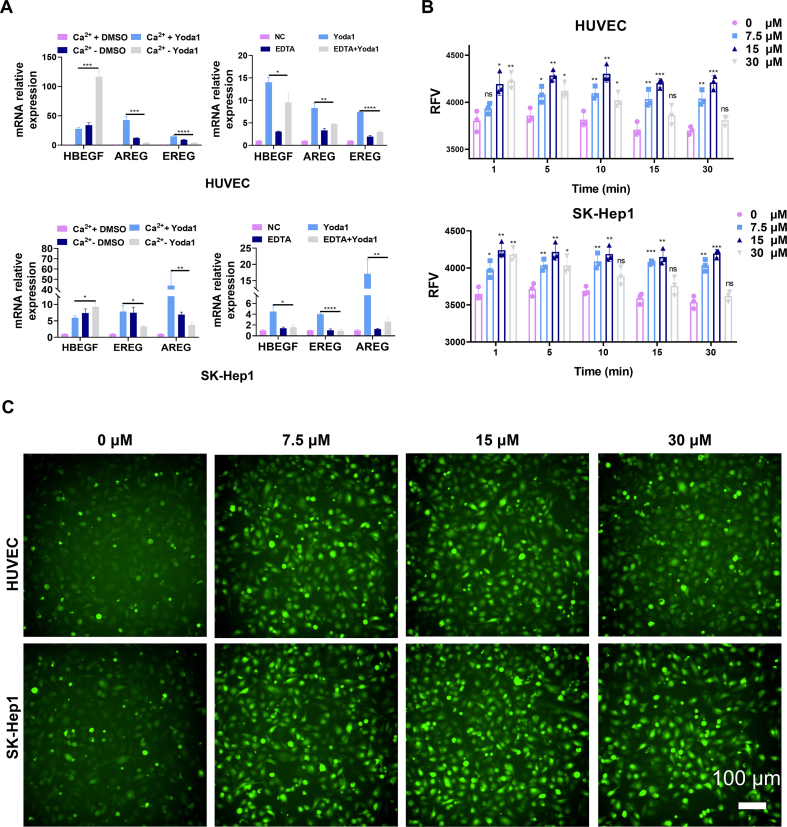


**figs8:**
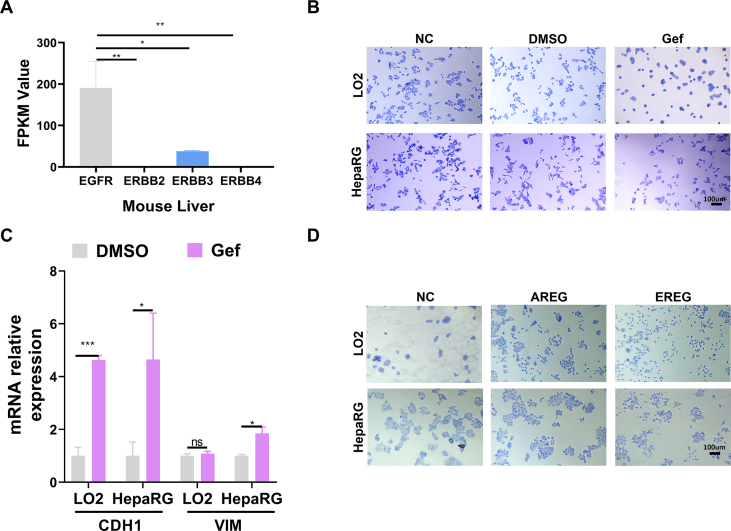


**figs9:**
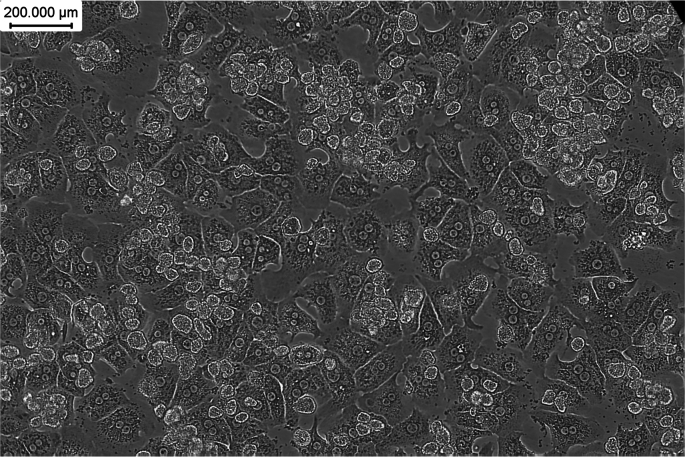


**figs10:**
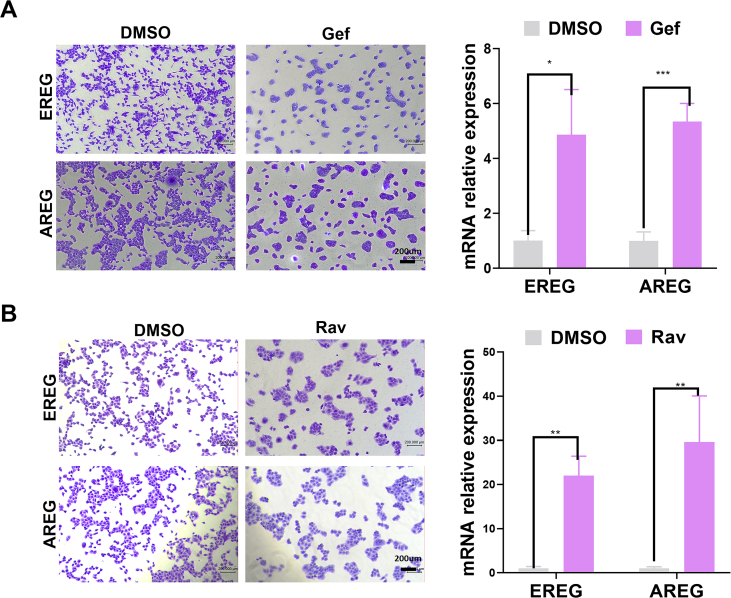


**figs11:**
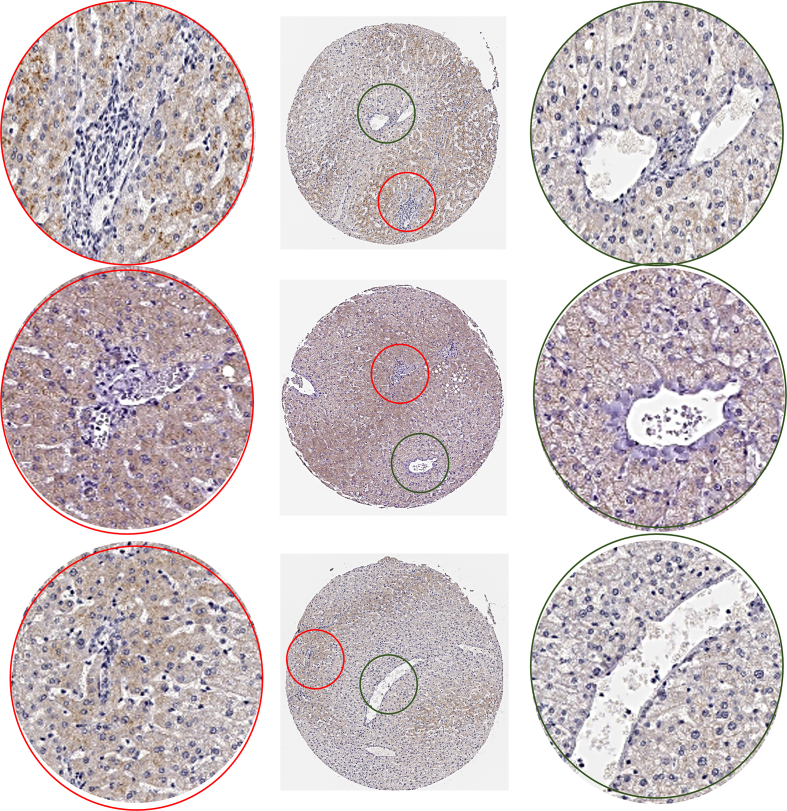


**figs12:**