# Supplementary material for: EGFR-mediated crosstalk between vascular endothelial cells and hepatocytes promotes Piezo1-dependent liver regeneration
Source: Genes Dis. 2024 May 8;12(3):101321. doi: 10.1016/j.gendis.2024.101321 (PMC11904541; doi:10.1016/j.gendis.2024.101321)
Supplement: Multimedia component 1 [file mmc1.docx]

**Supporting Information**


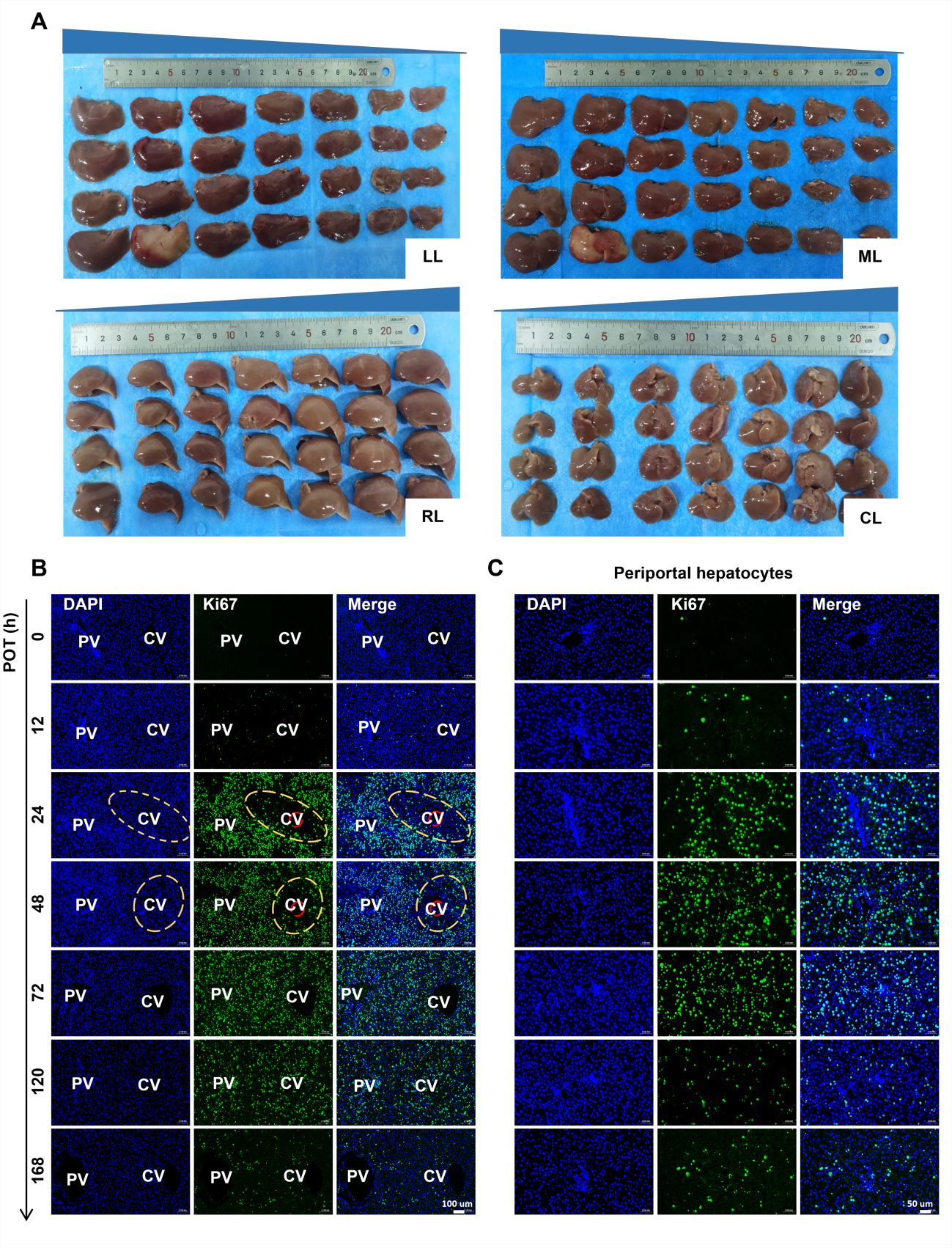


**Supplementary figure 1. PVL induced liver regeneration. (A)** Morphological changes of LL, ML, RL and CL of rat liver at different time points after 70% PVL. **(B)** Immunofluorescence staining analysis of Ki67 (in PV - CV) in paraffin tissues from right lobe liver at the different time points after 70% PVL. scale bar: 100 μm. **(C)** Immunofluorescence staining analysis of Ki67 (in PV zonation) in paraffin tissues from right lobe liver and at the different time points after 70% PVL . scale bar: 50 μm. PV: portal vine, LL: Left Lobe, ML: Middle Lobe, RL: Right Lobe, CL:Caudal Lobe, POT: post operative time.


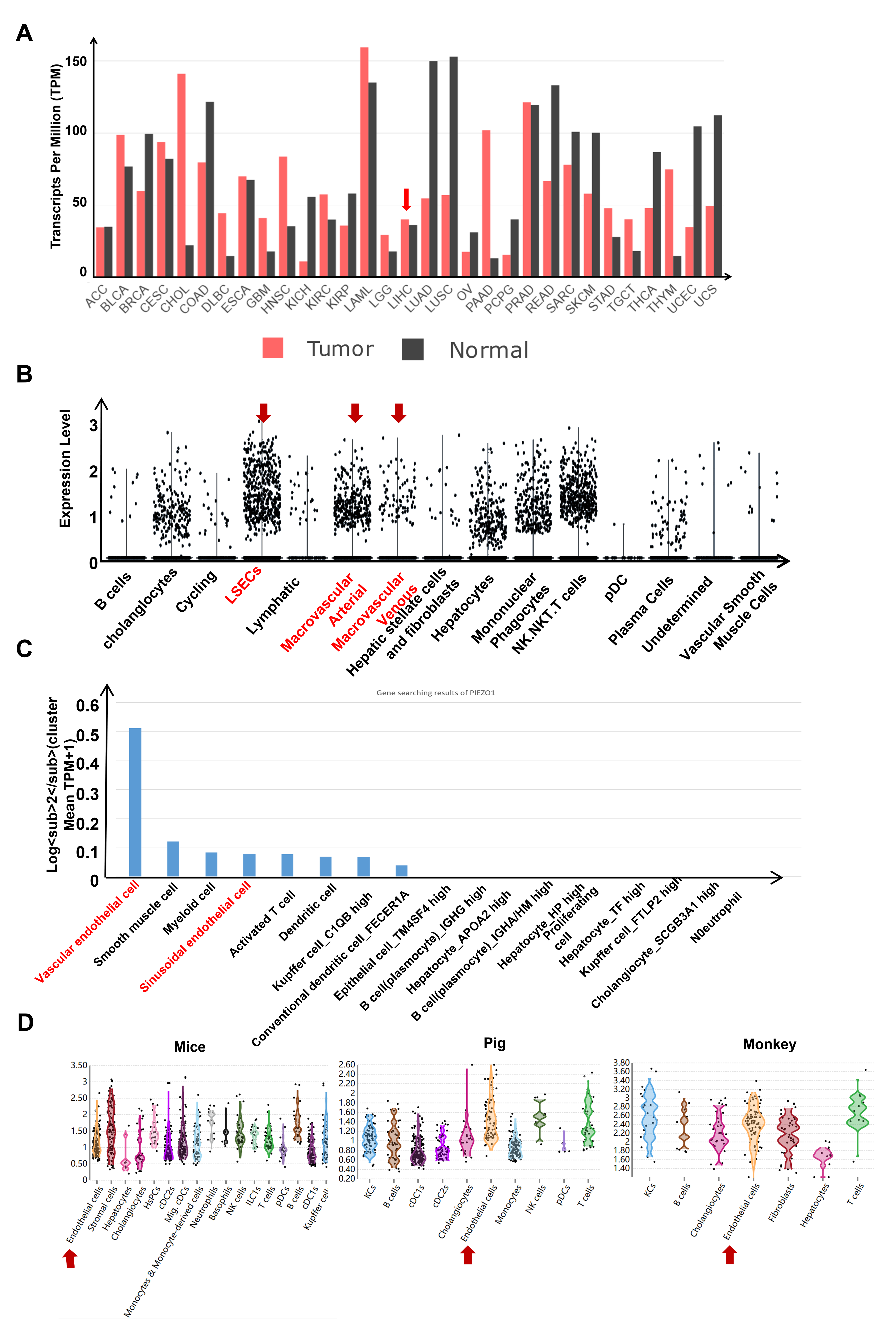


**Supplementary figure 2. The Piezo1 Expression levels in different cell types in the different public database. (A)** The GEPIA online platform analyzes suggest that Piezo1 expression in various tumor samples and paired normal tissues in the TCGA database. **(B** and **C)** Single-cell RNA sequencing revealed Piezo1 expression level in different cell subsets within the human liver (http://liveratlas-vilarinholab.med.yale.edu/ and <https://bis.zju.edu.cn/HCL/).> **(D)** Piezo1 expression within liver cell subsets of mice, pigs, and monkeys (https://livercellatlas.org).


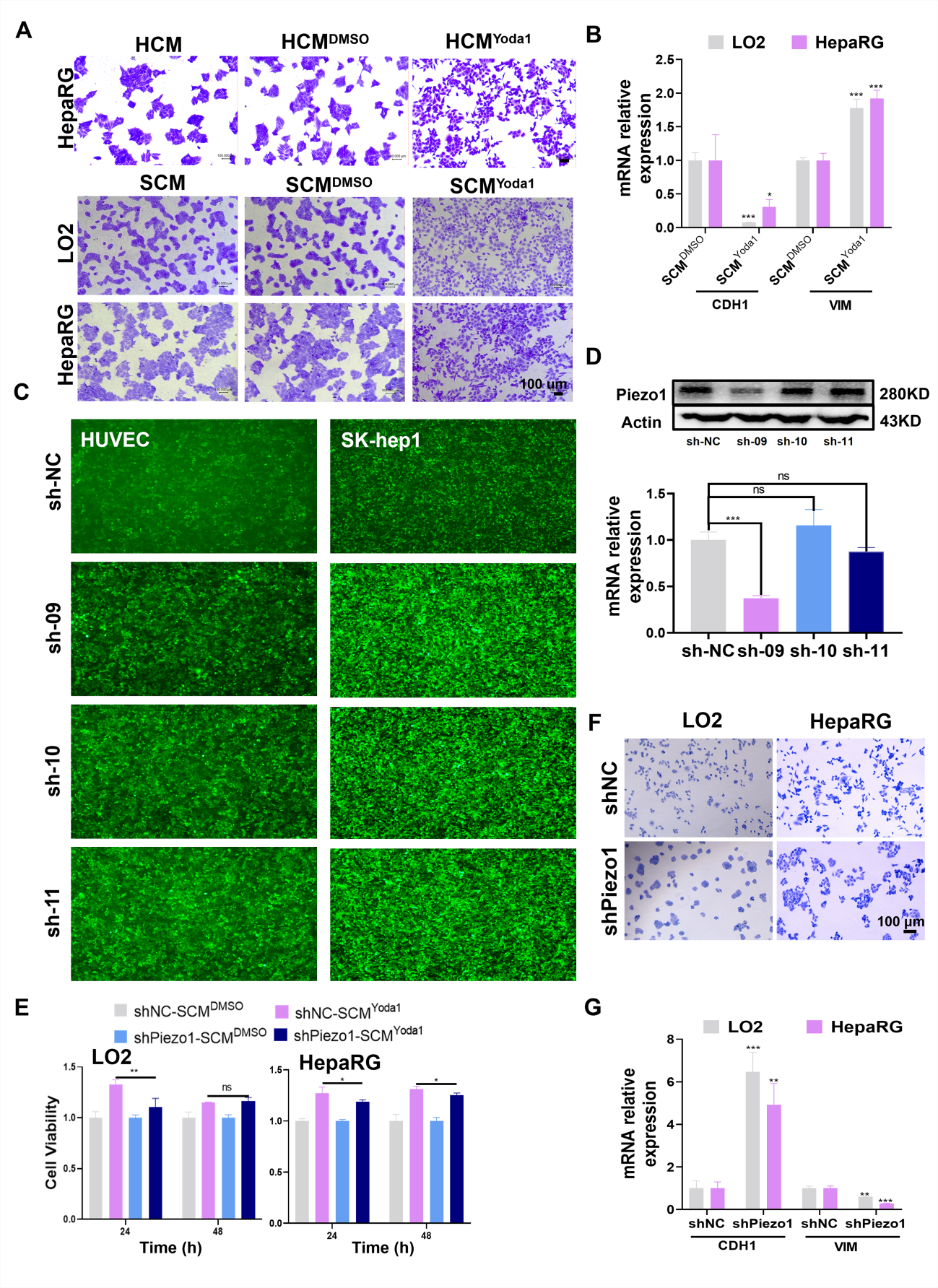


**Supplementary figure 3. Conditioned media from endothelial cells with Piezo1 activated by Yoda1 promotes hepatocyte proliferation.** **(A)** The representative morphology of hepatocytes cultured in CM of endothelial cells and morphological changes of hepatocytes induced by HCM-Yoda1 and SCM-Yoda1. **(B)** SCM-Yoda1 can promote EMT of hepatocytes and change of the mRNA expression level of CDH1 and VIM. **(C)** Fluorescence pictures of HUVEC and SK-hep1 stably transfected with shRNA. **(D)** WB and real-time quantitative PCR proved that shRNA knocks down the expression of *Piezo1* in SK-hep1. **(E** and **G)** Knockdown of *Piezo1* in endothelial cells can inhibit hepatocytes proliferation, morphological changes, and EMT induced by SCM-Yoda1. *, p < 0.05; **, p < 0.01; ***, p < 0.001; ****, p < 0.0001; two-tailed Student’s t tests. HCM^Yoda1^ and SCM^Yoda1^: Conditioned media from HUVEC and SK-hep1 with Piezo1 activated by Yoda1.


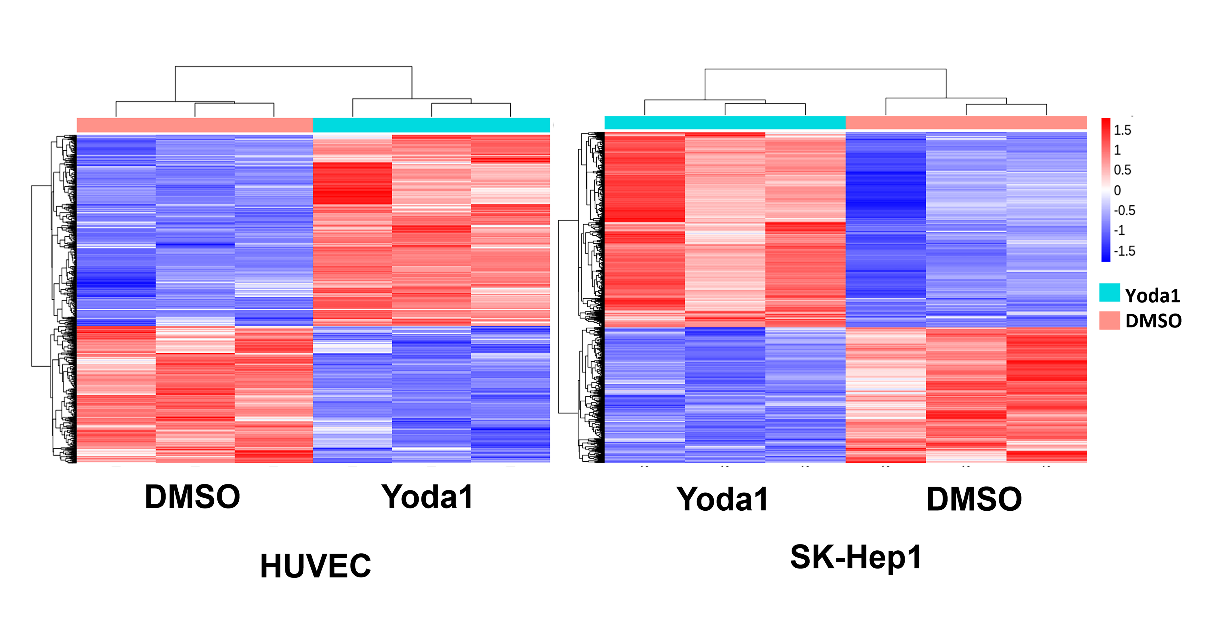


**Supplementary figure 4.** Two heatmaps show expression of differentially expressed genes between Yoda1 and DMSO treatment in HUVEC and SK-hep1 cell lines.


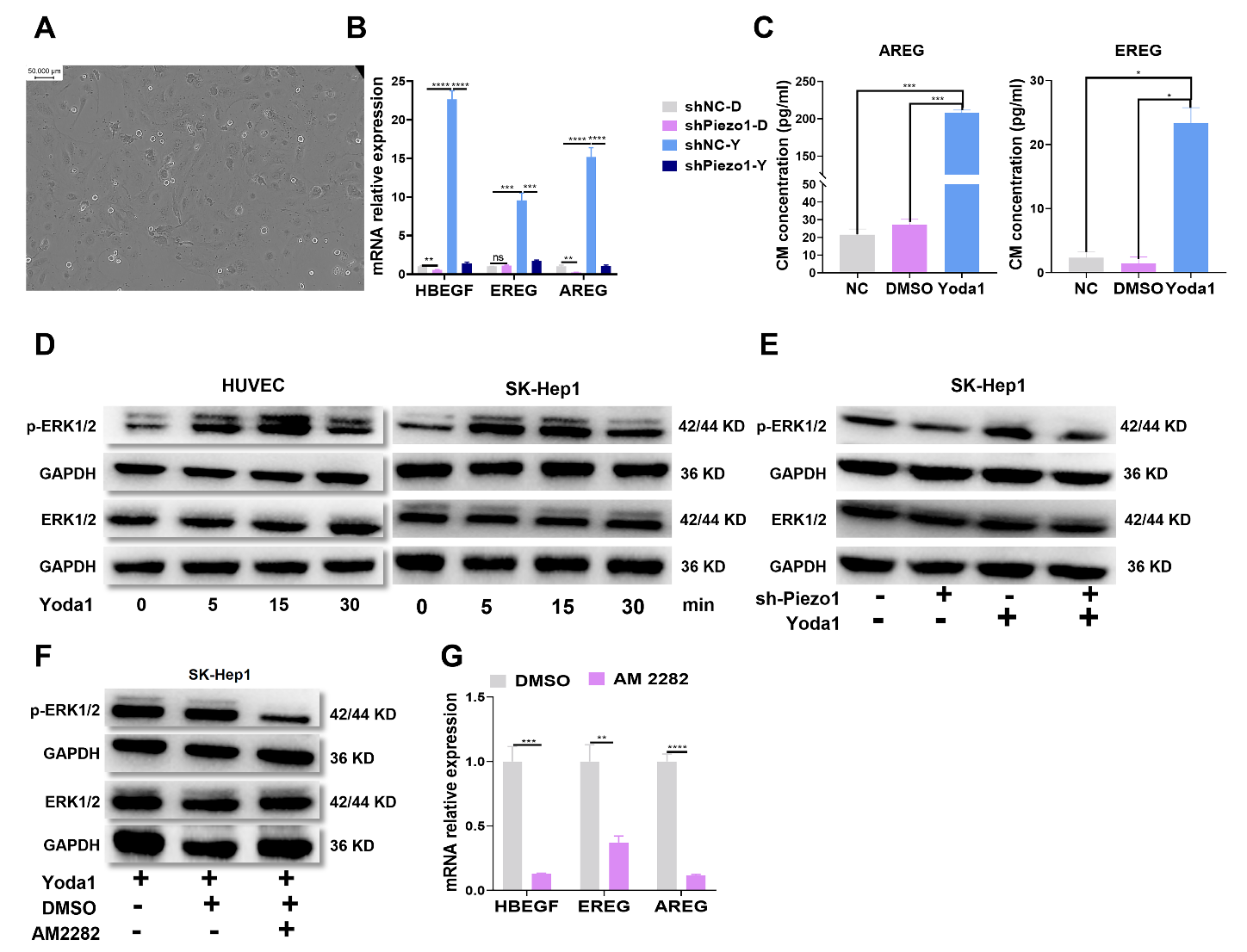


**Supplementary figure 5. The expression of HBEGF, EREG and AREG induced by Piezo1 activation in endothelial cells depends on PKC/ERK1/2 signaling pathway.** **(A)** Mouse primary vascular endothelial cells were isolated and cultured. **(B)** qRT-PCR demonstrated that the increased mRNA expression HBEGF, EREG and AREG induced by Yoda1 in SK-hep1 cells and were reversed with Piezo1 knock-down. **(C)** ELISA assay revealed that increased secretion of EREG and AREG in SCM-Yoda1. **(D** and **E)** Western blotting suggested that Yoda1 was able to induce enhanced ERK1/2 phosphorylation in HUVEC and SK-hep1 cells. **(F)** Western blotting confirmed that knockdown of *Piezo1* can inhibit the activation of ERK1/2 induced by Yoda1. **(G** and **H)** Western blotting and qRT-PCR demonstrated that non selective protein kinase inhibitor AM2282 (200 nM) can inhibit the activation of ERK1/2 and the expression of HBEGF, AREG and EREG in SK-hep1 cells induced by Yoda1. *, p < 0.05; **, p < 0.01; ***, p < 0.001; ****, p < 0.0001.


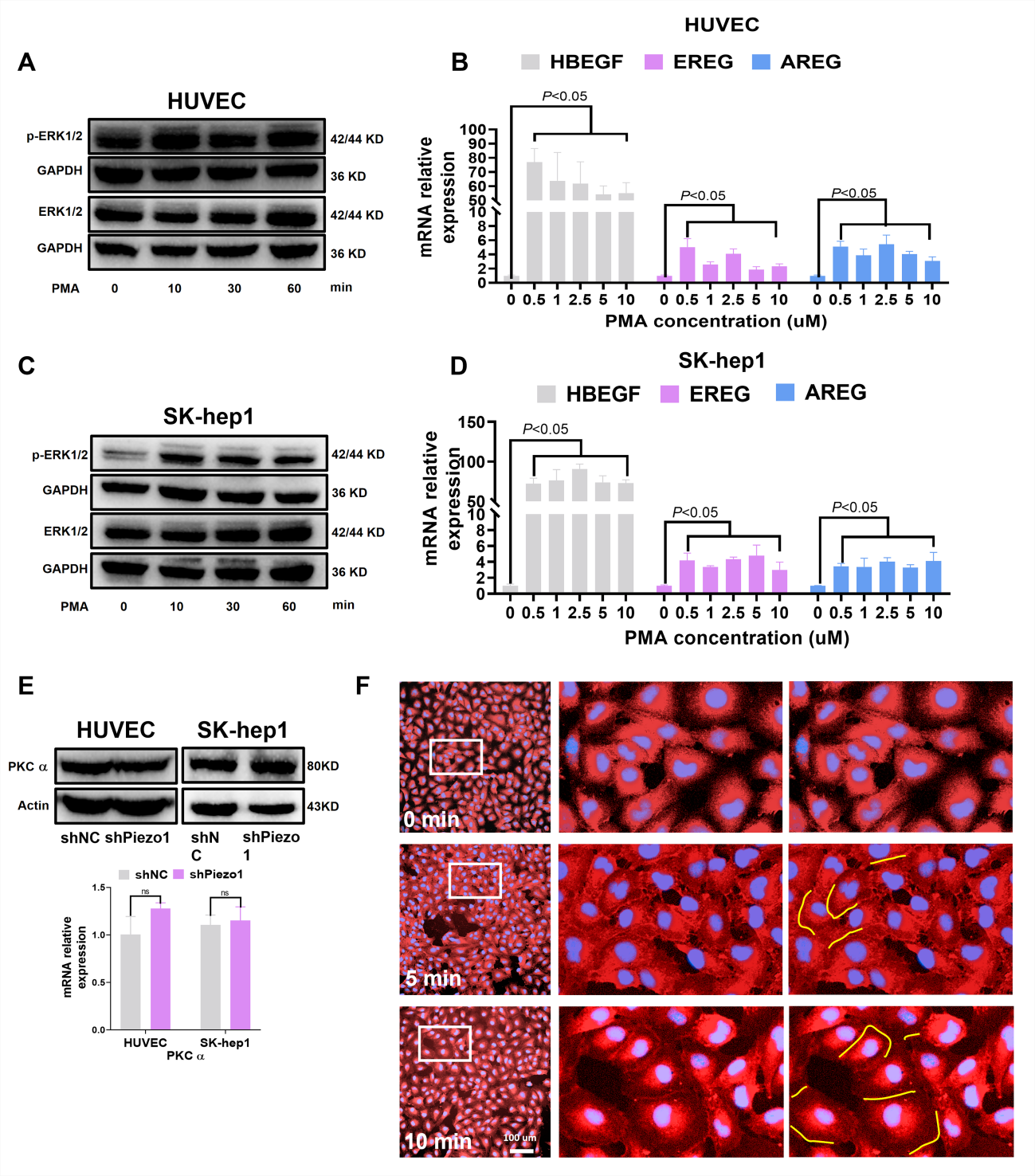


**Supplementary figure 6. The biological activity occurring in the endothelial cells after Piezo1 activation can be mimiced by PMA. (A-D)** Western blotting and qRT-PCR demonstrated that selective protein kinase C agonist PMA (WB:500nM) can enhance the activation of ERK1/2 and the expression of HBEGF, AREG and EREG in HUVEC cells. **(E** and **F)** Western blotting and qRT-PCR confirmed that knock-down of Piezo1 did not interfere the expression of PKCα. **(G)** Immunofluorescence analysis revealed that Yoda1(5μM and 5 min) activated PKCα. *, p < 0.05; **, p < 0.01; ***, p < 0.001; ****, p < 0.0001.

**
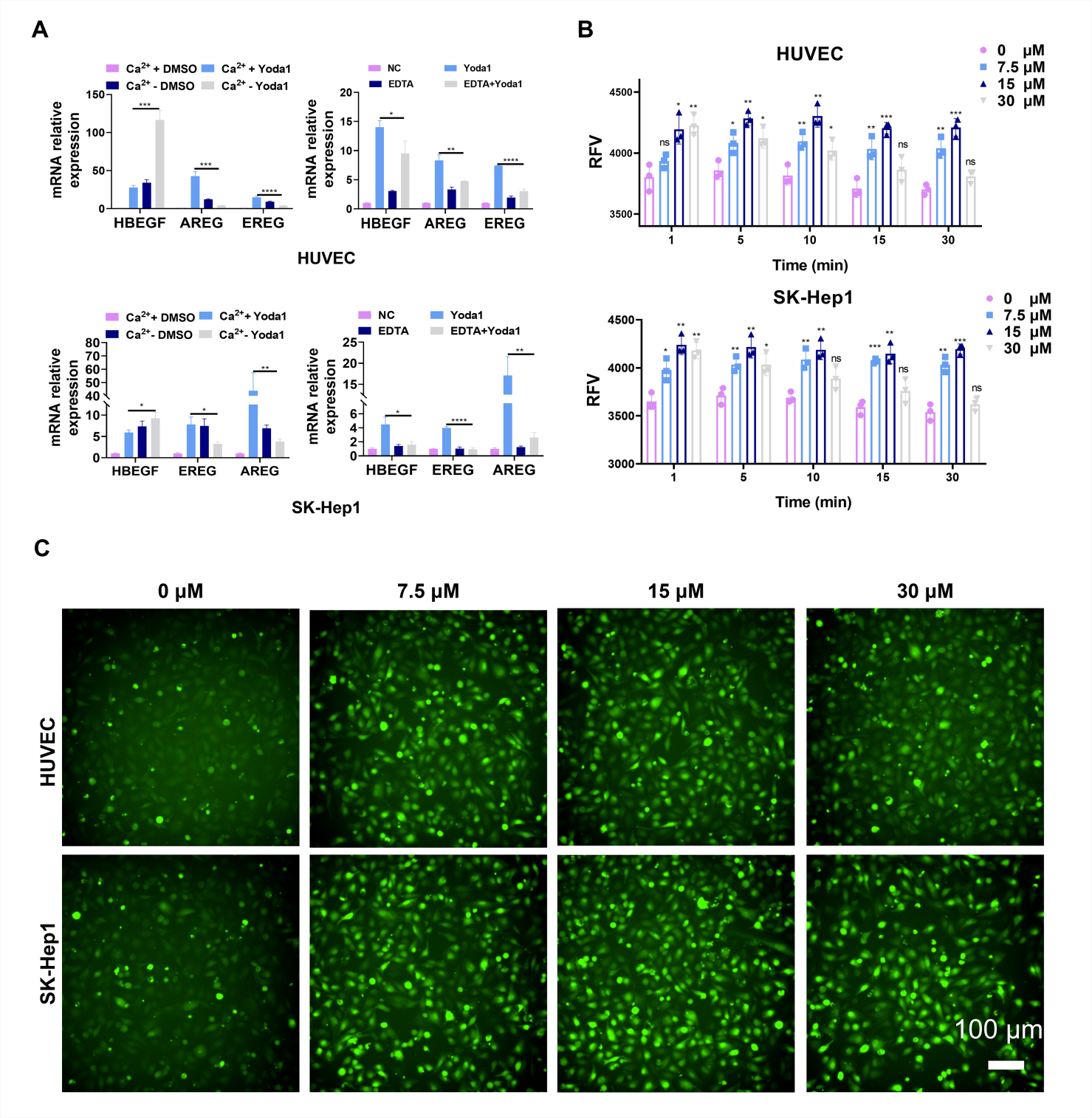
**

**Supplementary figure 7.** **The induction of HBEGF, EREG, and AREG expression in endothelial cells by Yoda1 is predominantly facilitated by calcium influx.** **(A)** We investigated the impact of a calcium-free medium and EDTA-mediated chelation of extracellular calcium ions on the Yoda1-induced expression of HBEGF, EREG, and AREG in HUVEC and SK-Hep-1 cells. **(B)** A fluorescence microplate reader was employed to measure the fluorescence intensity of HUVEC and SK-Hep-1 cells treated with Yoda1 at varying concentrations and time points (Yoda1: 0, 7.5, 15, 30 μM using the Fluo-4 calcium detection kit by Beyotime, The results of statistical analysis were obtained by comparing with the 0 μM Yoda1 group, respectively.). **(C)** A high-content imaging system quantified intracellular calcium levels in HUVEC and SK-Hep1 cells after 15 minutes of treatment with different concentrations of Yoda1 (Yoda1: 0, 7.5, 15, 30 μM, Fluo-4 calcium detection kit, Beyotime). RFV denotes relative fluorescence values. *, p < 0.05; **, p < 0.01; ***, p < 0.001; ****, p < 0.0001.


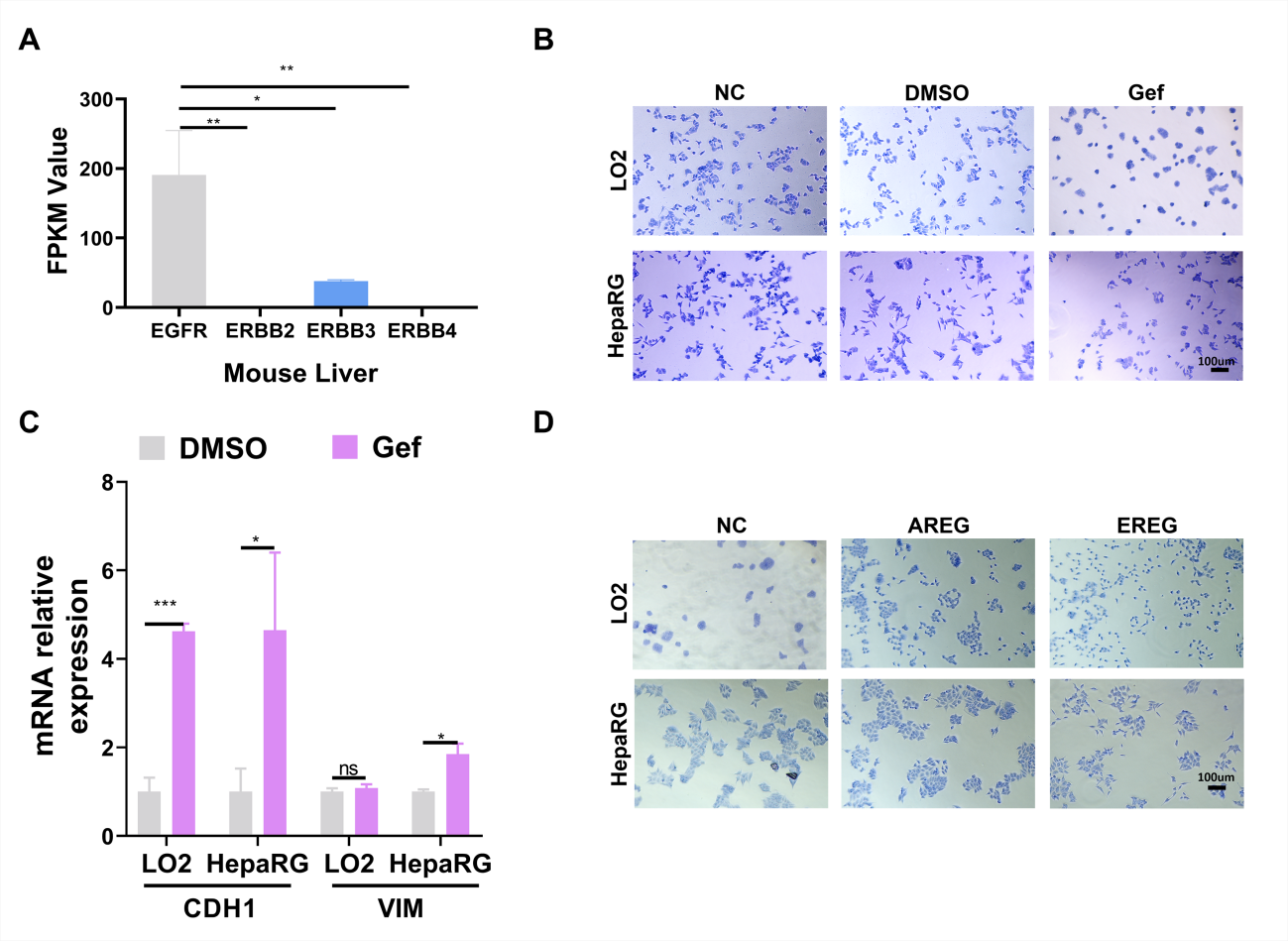


**Supplementary figure 8. AREG and EREG from endothelial cells promotes hepatocyte proliferation and epithelial loss through EGFR signal pathway**. **(A)** RNA sequencing revealed that the expression level of EGFR was highest in ERBB family within liver tissue (GEO data: 169242). **(B)** Crystal violet staining demonstrated that the morphological changes of hepatocytes induced by SCM^Yoda1^ were inhibited by gefitinib. **(C)** q-RT-PCR suggested that Gefitinib reverses the reduction of CDH1 induced by SCM^Yoda1^, but has no significant effect on VIM. **(D)** Crystal violet staining demonstrated that the morphological changes of hepatocytes induced by AREG and EREG. SCM^Yoda1^: Conditioned media from SK-hep1 with Piezo1 activated by Yoda1, *, p < 0.05; **, p < 0.01; ***, p < 0.001; ****, p < 0.0001.


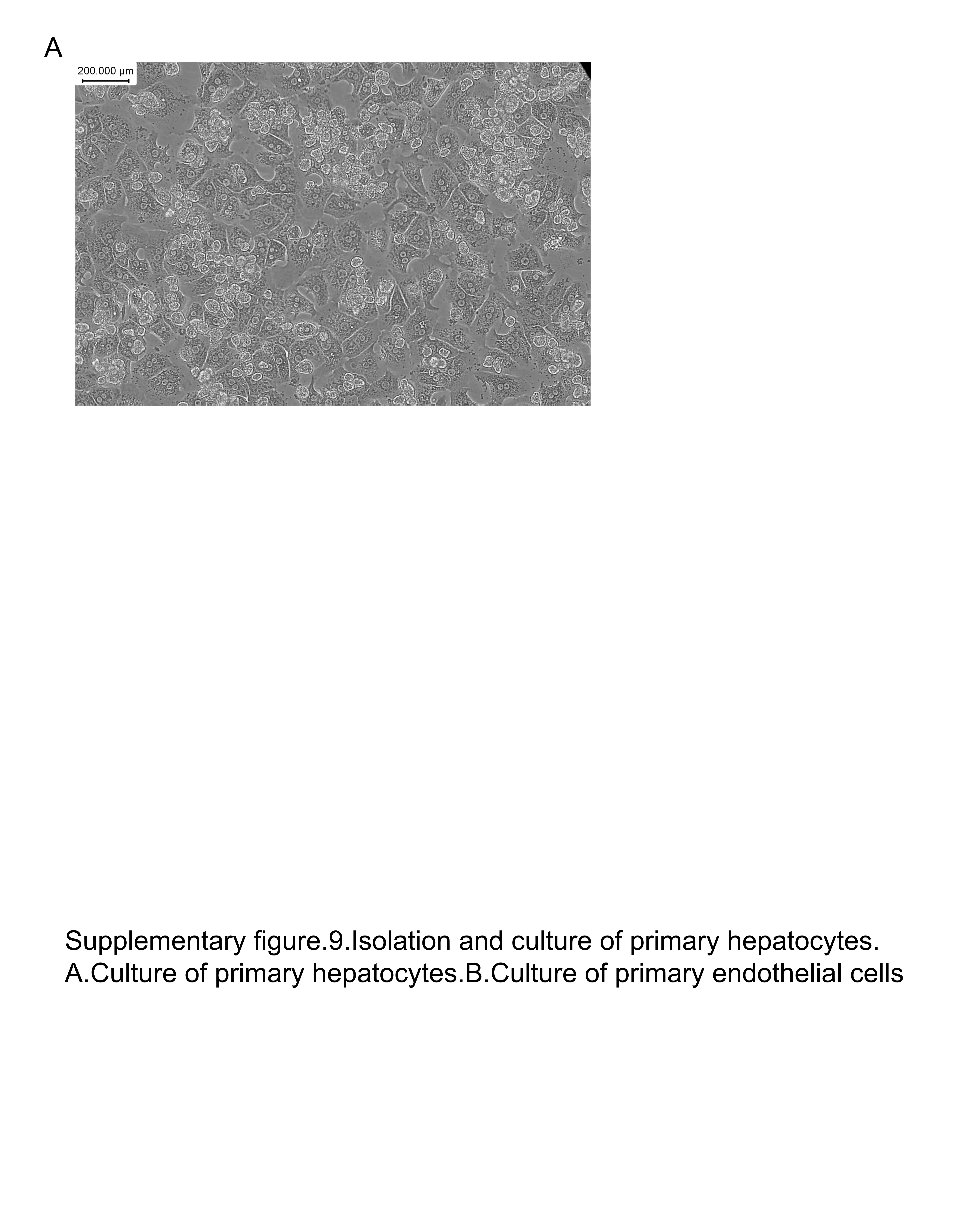


**Supplementary figure 9.** Isolation and culture of primary hepatocytes.


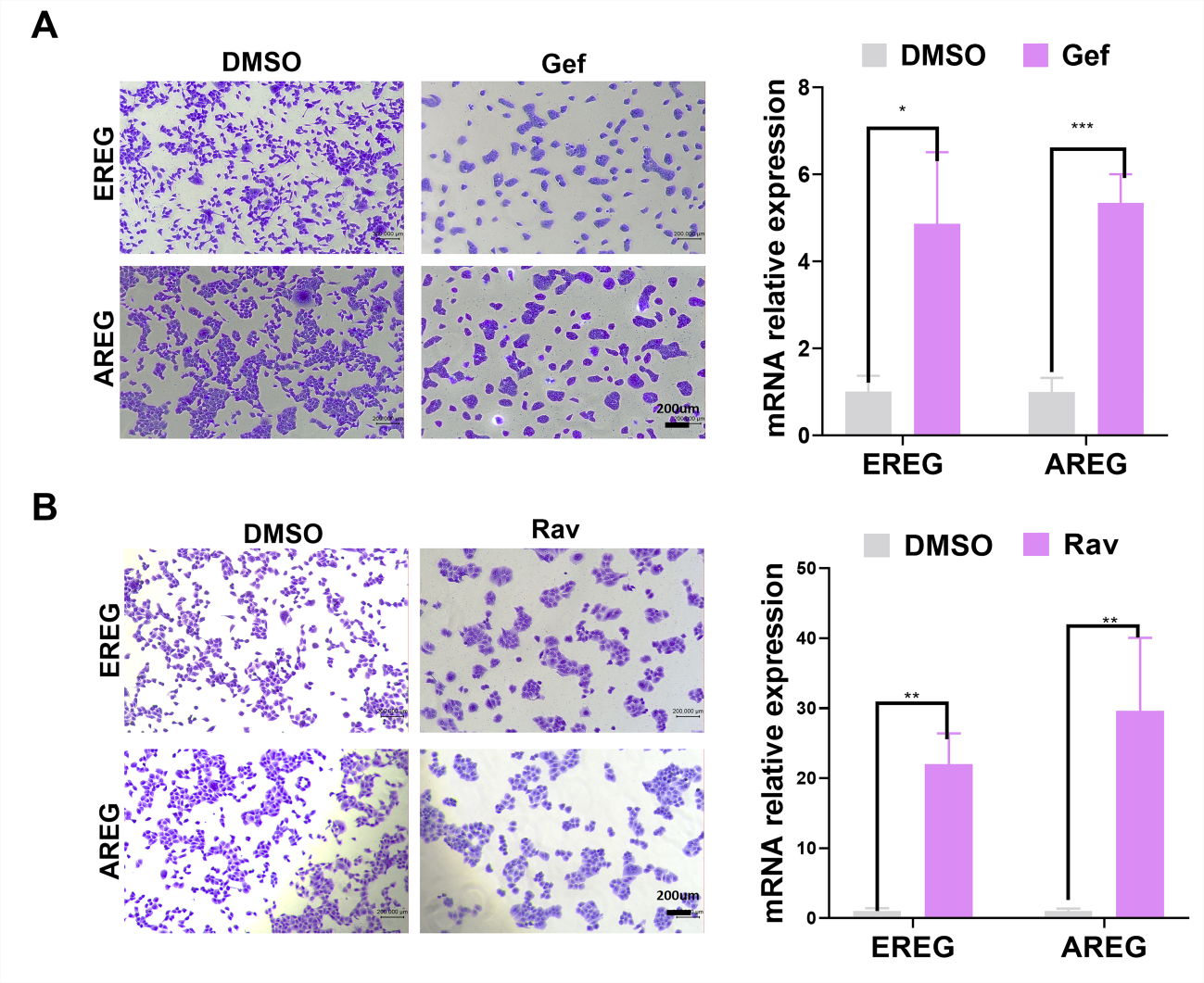


**Supplementary figure 10. EGFR is involved in both AREG-and EREG-induced hepatocyte partial EMT. (A** and **B)** Crystal violet staining and q-RT-PCR analysis proved that AREG and EREG promoted partial EMT (CDH1) of LO2. *, p < 0.05; **, p < 0.01; ***, p < 0.001; ****, p < 0.0001.


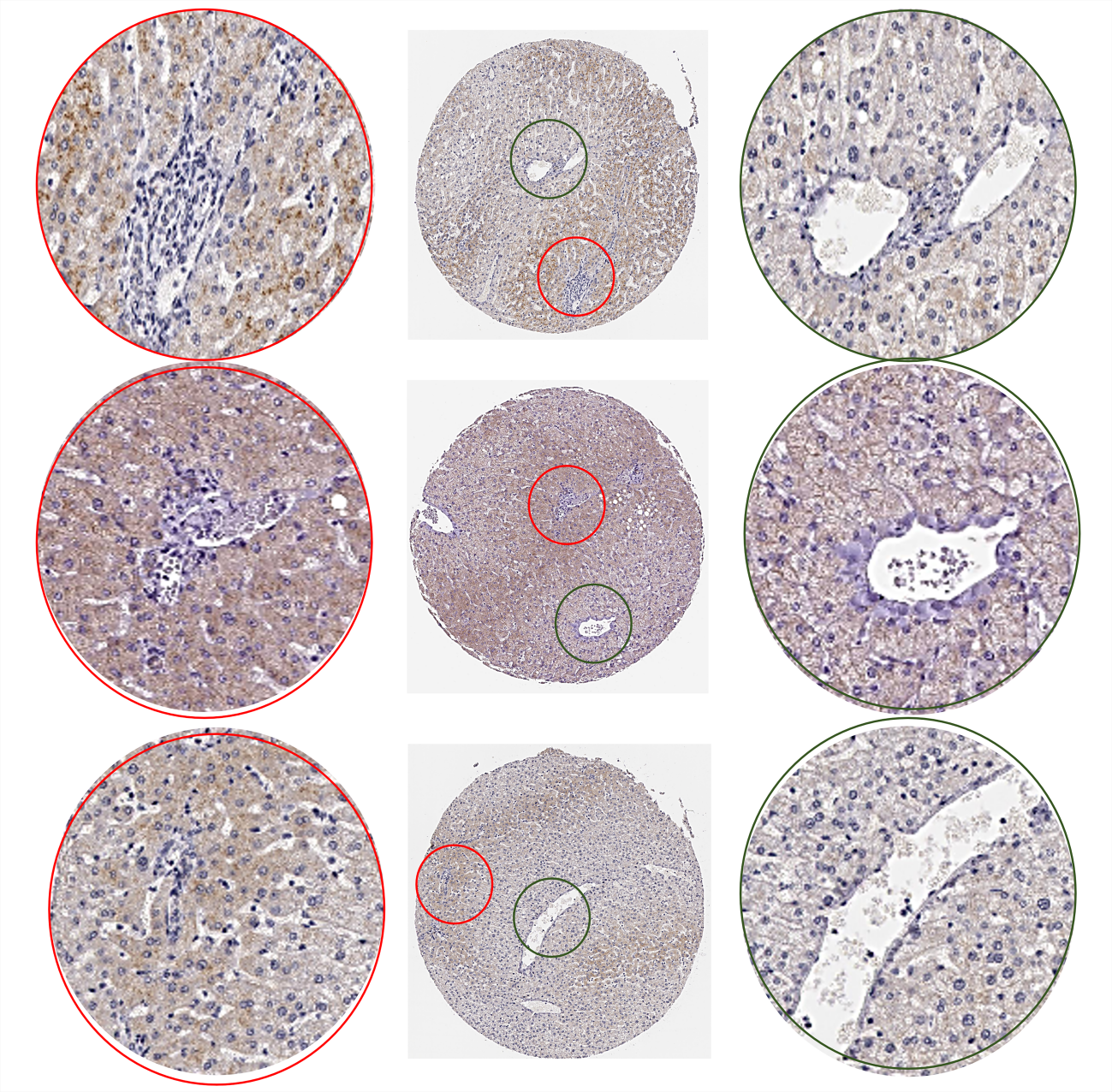


**Supplementary figure 11.** Immunohistochemistry results at the human protein atlas online site revealed higher expression of EGFR in hepatocytes in zones 1 and 2 than that in zone 3.


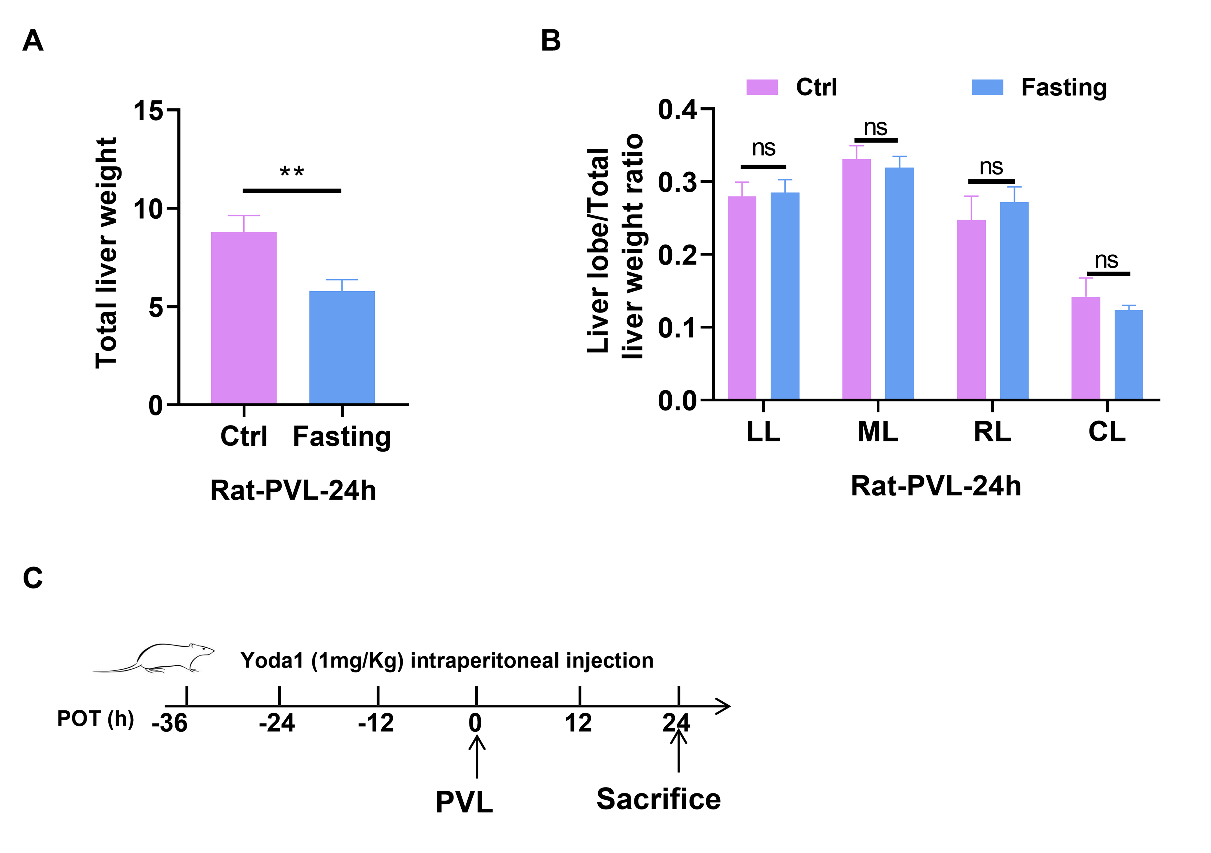


**Supplementary figure 12. The effect of fasting on liver weight and the application of Yoda1.** **(A)** The effect of fasting on the total liver weight of rats. **(B)** The effect of fasting on the proportion of liver lobes. **(C)** The schematic diagram of Yoda1 application scheme.

Supplementary table 1. The formulation of solutions 1-3.

| Solution | formulation | | |
| --- | --- | --- | --- |
| Solution1 （S1） |  | | |
|  | NaCl | 9.0g | 1L water |
|  | hepes | 4.76g |  |
|  | KCl | 0.42g |  |
|  | Glucose | 0.9g |  |
|  | NaHCO3 | 2.1g |  |
| Solution2(S2) |  | | |
|  | EGTA (100mM) | 10ml | 990ml S1 |
| Solution2(S2) |  | | |
|  | Cacl2·2H2O (0.5mM) | 10ml | 990ml S1 |

Supplementary table 2. List of primer sequences used for q-RT-PCR

| Gene name | Forward | Reverse |  |
| --- | --- | --- | --- |
| HBEGF | TCATGTTTAGGTACCATAGGAGAG | AGTGGGAATTAGTCATGCCC | Human |
| Piezo-1 | CTCTTCCTGGCGCTGTTC | GATGAGGTTGGTGGAGTTGG |  |
| β-Actin | CCTGGCACCCAGCACAAT | GGGCCGGACTCGTCATAC |  |
| AREG | GAGCCGACTATGACTACTCAGA | TCACTTTCCGTCTTGTTTTGGG |  |
| EREG | ATGTGGCTTTGACCGTGATTC | TCCCCTGAGGTAACTCTCTCATA |  |
| NRG1 | CGGTGTCCATGCCTTCCAT | GGGAGGCTGTTACTGTCATGC |  |
| PRKCA | GTCCACAAGAGGTGCCATGAA | AAGGTGGGGCTTCCGTAAGT |  |
| CDH1 | AAAGGCCCATTTCCTAAAAACCT | TGCGTTCTCTATCCAGAGGCT |  |
| CDH2 | AGCCAACCTTAACTGAGGAGT | GGCAAGTTGATTGGAGGGATG |  |
| VIM | TGCCGTTGAAGCTGCTAACTA | CCAGAGGGAGTGAATCCAGATTA |  |
| m-GAPDH | TGGCCTTCCGTGTTCCTAC | GAGTTGCTGTTGAAGTCGCA | Mouse |
| m-CDH1 | CAGTTCCGAGGTCTACACCTT | TGAATCGGGAGTCTTCCGAAAA |  |
| m-VIM | CGTCCACACGCACCTACAG | GGGGGATGAGGAATAGAGGCT |  |
| m-HBEGF | CGGGGAGTGCAGATACCTG | TTCTCCACTGGTAGAGTCAGC |  |
| m-AREG | GGTCTTAGGCTCAGGCCATTA | CGCTTATGGTGGAAACCTCTC |  |
| m-EREG | TTGGGTCTTGACGCTGCTTT | TGCATGATGGGATCACGGTTG |  |
